# Inferring multi-target QSAR models with taxonomy-based multi-task learning

**DOI:** 10.1186/1758-2946-5-33

**Published:** 2013-07-11

**Authors:** Lars Rosenbaum, Alexander Dörr, Matthias R Bauer, Frank M Boeckler, Andreas Zell

**Affiliations:** 1 Center for Bioinformatics (ZBIT), University of Tübingen, Sand 1, Tübingen 72076, Germany; 2 Institute of Pharmaceutical Sciences, University of Tübingen, Auf der Morgenstelle 8, Tübingen 72076, Germany

**Keywords:** Proteochemometrics, QSAR, Multi-target, Support vector machine, Kinome, Machine learning, Multi-task, Domain adaption

## Abstract

**Background:**

A plethora of studies indicate that the development of multi-target drugs is beneficial for complex diseases like cancer. Accurate QSAR models for each of the desired targets assist the optimization of a lead candidate by the prediction of affinity profiles. Often, the targets of a multi-target drug are sufficiently similar such that, in principle, knowledge can be transferred between the QSAR models to improve the model accuracy. In this study, we present two different multi-task algorithms from the field of transfer learning that can exploit the similarity between several targets to transfer knowledge between the target specific QSAR models.

**Results:**

We evaluated the two methods on simulated data and a data set of 112 human kinases assembled from the public database ChEMBL. The relatedness between the kinase targets was derived from the taxonomy of the humane kinome. The experiments show that multi-task learning increases the performance compared to training separate models on both types of data given a sufficient similarity between the tasks. On the kinase data, the best multi-task approach improved the mean squared error of the QSAR models of 58 kinase targets.

**Conclusions:**

Multi-task learning is a valuable approach for inferring multi-target QSAR models for lead optimization. The application of multi-task learning is most beneficial if knowledge can be transferred from a similar task with a lot of in-domain knowledge to a task with little in-domain knowledge. Furthermore, the benefit increases with a decreasing overlap between the chemical space spanned by the tasks.

## Background

Much has happened in the process of rational drug discovery in the last decades. The technology of next-generation sequencing [1] with its possibility to sequence genomes in an accelerating pace pushed the door open to a new set of targets approachable by existing and future drugs. Additionally, the methods of combinatorial chemistry [2] enable pharmaceutical chemists to generate large compound libraries by synthesizing more and more drug-like molecules. To process these enormous amounts of data, advances in the field of high-throughput screening complement the previously mentioned methods in a way that an increasing number of compounds can be screened against desired biological targets with a decreasing financial effort [3]. Regarding these facts and looking at the increased amount of R&D investments, one could argue that the drug discovery pipeline should be in full swing yielding a growing amount of approved drugs. Albeit, the number of novel drugs did not increase but rather, if any, stayed constant [4].

A joint starting point of many drug design approaches is an exhausting search for a drug-like molecule that binds with a high affinity to a desired biological target. However, recent findings have shown that looking for such a high affinity binder for a specific receptor is not crowned with success in every case. Even if single-target drugs can evoke the pursued effect on their specific biological target, this does not necessarily apply to the whole organism [5,6]. For example the targets associated with the treatment of complex diseases like impairment of the CNS, cancer, metabolic disorders, or AIDS are diverse and several disease related mechanisms have to be taken into account [7,8]. Targeting multiple proteins is required for these diseases because medication of the diseased state is intercepted by the way the proteins interact such that back-up circuits or fail-safe mechanisms take effect. These backup systems can be sufficiently dissimilar that they do not respond to a highly selective drug [8-11]. Hence, in cancer therapy, drugs with a single or few targets can be doomed to failure, since resistances are more easily to arise than if pressure is exerted on more targets [12].

In addition to new ways of treating diseases like cancer, the approach of multi-target drug design offers various advantages. Using a single molecule for different pathways in a chemotherapy increases its therapeutic effectiveness, and it is much easier to manage absorption and elimination for one molecule than for several [13]. Compared to single-target drugs that bind with a high affinity to their target, multi-target drugs are considered low-affinity binders [6]. From this fact it follows that multi-target drugs are not subject to the high constraints for high-affinity binding and, furthermore, allow for targeting a greater number of proteins [8]. In some cases, like the operation of NMDA receptor antagonists, it is in fact desirable to bind with a lower affinity, since shutting this receptor completely down is impairing its normal function [14,15]. There is also evidence that several small interventions to various targets, as achieved with multi-target drugs, can have a greater effect on the outcome than a strong single perturbation [6,16].

The multi-target drug design approach is a promising way to complement the existing single-target process and a plethora of studies address the problem of target prediction [17] and multi-target structure-activity models [18-20]. Ma et al. [18] evaluated support vector machine (SVM) classification models of several biological targets for common hits. Heikamp et al. [19] linearly combined independently derived SVM models by assigning a distinct weight to each model. Ajmani et al. [20] inferred models for three kinases with PLS regression methods and evaluated the models for common structural requirements to inhibit the kinases. These studies show that multi-target drug prediction is a contemporary research topic in the field of drug design. Despite the positive results of the studies mentioned above, the considered models were still trained for each target separately.

Studies in the field of multi-task and transfer learning suggested a promising way to combine knowledge from problem-related tasks into a single SVM model. Schweikert et al. [21] argued that from the kinship of organism one can see analogous biochemical processes. Therefore, it is possible to transfer the knowledge of a biological problem to another domain if both problems are sufficiently related to each other. This domain adaption approach was successfully applied to the binding prediction of MHC class I molecules and splice site detection [22]. Looking beyond the lead identification process and with it the classification of molecules, support vector regression (SVR) can be utilized to reveal and address the specific affinity of molecules during the optimization of potential drugs. Developing a multi-target agent requires to monitor the affinity against a panel of similar targets. Thus, adapting multi-task classification to a regression setting should be beneficial for the lead optimization of multi-target drugs. Multi-target regression algorithms can compensate for a fewer amount of training instances available for a problem by exploiting the knowledge of a similar problem.

The concept of taxonomy-based transfer learning is similar to the concept of overlapping ligand–target spaces in the field of proteochemometric modeling. A proteochemometric model is trained on instances that combine target descriptors with ligand descriptors. An overview of proteochemometrics can be found in a recent review by van Westen et al. [23]. In contrast to proteochemometric models, transfer learning algorithms infer target specific models solely on ligand descriptors, but force the models to be similar according to some target similarity or taxonomy.

In this paper, we present two different multi-task regression algorithms based on the multi-task classifiers of Widmer et al. [22]. We demonstrate the effectiveness of the algorithms by inferring multi-target QSAR models on a subset of the human kinome. The taxonomical relationship of the kinase targets should correlate with the relatedness of the QSAR problems on these targets. Hence, we derived the relatedness of the problems from the human kinome tree [24]. We compared our multi-task methods to SVM models that were independently trained for each target and an SVM model that assumed all targets to be identical. We evaluated the methods on simulated data sets, a data set with affinity data against a large fraction of the human kinome, and four smaller subsets of the aforementioned kinome data.

The results show that multi-target learning results in a considerable performance gain compared to the baseline methods if knowledge can be transferred from a target with a lot of data to a similar target with little domain knowledge.

## Methods

First, this section shortly recaps standard support vector regression. Second, we present two multi-task learning approaches that can be used for multi-target QSAR and discuss how they can be parametrized. Finally, we shortly explain the employed molecular encoding and the baseline methods used for comparison.

### Standard support vector regression (SVR)

**Figure 1:**
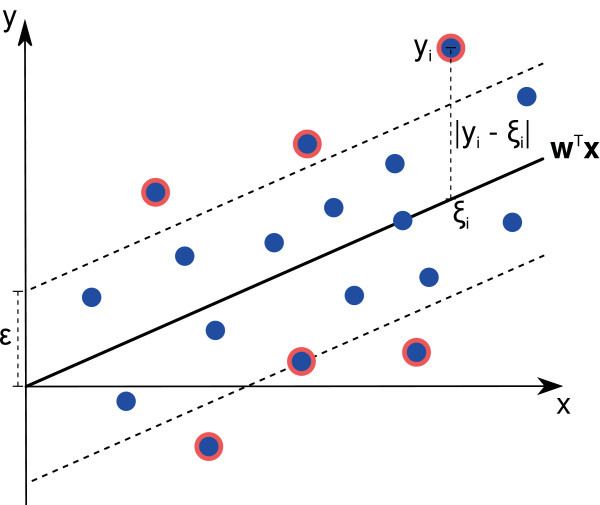
**Support vector regression (SVR).** Illustration of an SVR regression function represented by **w**^*T*^**x**. The *∊*-insensitive tube around the function is indicated by a gray tube. *ξ*_*i*_ = **w**^*T*^*x*_*i*_ is the predicted target value of *x*_*i*_ and *y*_*i*_ represents the actual target value. Support vectors are indicated by a red border.

A single-target QSAR problem comprises a set of *l* labeled fingerprints $\left\{(x_{i},y_{i}),i=1,\ldots ,l\right\}$, where $x_{i}\in {\mathbb{R}}^{n}$ is a fingerprint of a compound and $y_{i}\in \mathbb{R}$ is a pIC50 or pKi value. Given such a QSAR data set the standard support vector regression (SVR) solves the constrained optimization problem shown in Equation 1, which is also known as primal problem. A visualization of the problem’s variables is presented in Figure 1.

(1) $$\begin{array}{ll} \underset{w,\xi }{\text{min}} & \;\frac{1}{2}||w|{|}^{2}+C\overset{l}{\underset{i=1}{\sum }}l_{∊}(\xi_{i},y_{i})\\ \mathrm{s.t.} & \;\xi_{i}=w^{T}x_{i} \end{array}$$

In Equation 1, the term ||**w**||^2^ regularizes the model complexity, *C*>0 is a user-defined parameter and the *∊*-insensitive loss function *l*_*∊*_ is defined as follows.

(2) $$l_{∊}(\xi_{i},y_{i})=\left\{\begin{array}{ll} \text{max}(|\xi_{i}-y_{i}|-∊,0) & \text{or}\\ \text{max}{\left(|\xi_{i}-y_{i}|-∊,0\right)}^{2} \end{array}\right)$$

The function *l*_*∊*_ ensures that the loss is zero if |**w**^*T*^*x*_*i*_−*y*_*i*_|=|*ξ*_*i*_−*y*_*i*_|≤*∊*, which means that the actual target value *y*_*i*_ lies within an *∊*-insensitive tube around **w**^*T*^**x**. Equation 2 is commonly known as L_1_ and L_2_ SVR loss, respectively. In this study, we use the mean squared error (MSE) as error function, which is directly modeled by the L_2_ loss. Hence, the equations throughout the paper assume that L_2_ loss is applied.

The dual problem *f*_*D*_(***β***) of L_2_ loss SVR is presented in Equation 3, where *Q*_*ij*_=*x*_*i*_^*T*^*x*_*j*_ is the so called kernel matrix.

(3) $$\begin{array}{ll} \underset{\beta }{\text{min}} & \;f_{D}(\beta )=\\ \underset{\beta }{\text{min}} & \;\frac{1}{2}\beta^{T}Q\beta +\overset{l}{\underset{i=1}{\sum }}\left(\;∊|\beta_{i}|-y_{i}\beta_{i}+\frac{1}{4C}\beta_{i}^{2}\right) \end{array}$$

The data points, for which *β*_*i*_≠0, are called support vectors. A data point is a support vector if and only if its actual target value *y*_*i*_ is on the boundary or outside the *∊*-insensitive tube around the predicted value **w**^*T*^*x*_*i*_. The larger the value of *∊*, the sparser the resulting SVR model, but the less precise the model needs to approximate the target values *y*_*i*_. For the derivation of the dual problem and a more detailed introduction to SVR theory, we refer to [25,26].

The dual problem (3) can be rapidly solved with the large-scale learning library LIBLINEAR [27]. The library uses a dedicated solver [26], which allows for training an SVR model with several hundred thousands of instances. However, the library is limited to the linear case, which means that the dot product kernel has to be used.

Generally, the dot product kernel results in larger similarity values with an increasing compound or fingerprint size. Hence, we normalize each fingerprint before training, such that ∥*x*_*i*_∥=1. This normalization in combination with the dot product kernel is equal to using the cosine kernel as shown in Equation 4.

(4) $$k_{\text{cos}}(x_{i},x_{j})=\frac{{x_{i}}^{T}x_{j}}{∥x_{i}∥∥x_{j}∥}$$

The similarity values of the cosine kernel are normalized to [ 0,1] and are independent of the fingerprint size. As a result, the cosine kernel generally performs better for chemical fingerprints than using the dot product kernel without normalization.

### Multi-task learning

A multi-target QSAR data set with *T* different targets comprises a set of triples $\left\{(x_{i},y_{i},t_{i}),i=1,\ldots ,l\right\}$, where *x*_*i*_ and *y*_*i*_ are defined as for a single-target QSAR problem, and $t_{i}\in \{1,\ldots ,T\}$ indicates to which target protein the triple belongs to. For multi-target QSAR, inferring the QSAR model for a certain target *t* can be regarded as a separate learning task.

The goal of multi-task learning is to learn a set of functions $f_{T}$ such that $f_{t_{i}}(x_{i})\approx y_{i}$ and the set $f_{T}$ generalizes well to unseen data. Multi-task learning belongs to the field of transfer learning. In transfer learning, knowledge of a well known domain *s* is transferred to a similar, less known domain *t*. By transferring knowledge, the resulting function *f*_*t*_ should generalize better on unseen data. Consequently, transfer learning should be most profitable if a learning task with very few training instances is similar to a learning task with many training instances.

The knowledge transfer is commonly achieved by forcing the functions *f*_*s*_ and *f*_*t*_ to be similar if the domains *s* and *t* are similar. For linear SVR models, a function *f*_*t*_(**x**)=*w*_*t*_^*T*^**x** is completely determined by its weight vector *w*_*t*_. The weights $w_{1},\ldots ,w_{T}$ are forced to be similar by changing the SVR primal (1) to Equation 5.

(5) $$\begin{array}{l} \underset{w_{1},\ldots ,w_{T},\xi }{\text{min}}\;\frac{1}{2}\overset{T}{\underset{t=1}{\sum }}||w_{t}|{|}^{2}+J(w_{1},\ldots ,w_{T})\\ \;+C\overset{l}{\underset{i=1}{\sum }}l_{∊}(\xi_{i},y_{i})\\ \;\xi_{i}={w_{t_{i}}}^{T}x_{i} \end{array}$$

**Figure 2:**
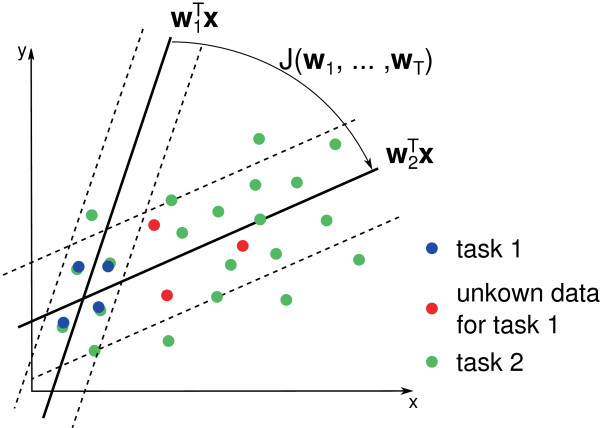
**Knowledge transfer in multi-task learning.** Illustration of a knowledge transfer from task 2, which comprises a lot of training data (green), to a similar task 1, which contains little training data (blue). The *∊*-insensitive tubes around the regression functions *w*_1_^*T*^**x** and *w*_2_^*T*^**x** are colored gray. The regularizer $J(w_{1},\ldots ,w_{T})$ forces the model of task 1 (*w*_1_) to be more similar to the model of task 2 (*w*_2_). A model *w*_1_ that is more similar to *w*_2_ predicts the unknown data (red) better, which results in a better generalization of the model.

The terms ||*w*_*t*_||^2^ control the task specific model complexity, like for standard SVR. The function $J(w_{1},\ldots ,w_{T})$ represents an additional regularization term that facilitates the similarity of the weight vectors of similar tasks. The type of multi-task learning algorithm is determined by a specific choice of the regularizer $J(w_{1},\ldots ,w_{T})$[28-31]. An example on how multi-task learning transfers knowledge between tasks is depicted in Figure 2.

Given an unseen data point **x**, the target value *y* for a specific task *t* can be obtained by *f*_*t*_ as shown in Equation 6.

(6) $$y=f_{t}(x)={w_{t}}^{T}x$$

A task specific bias term *b*_*t*_ can be included in the training and in the decision function by adding the bias to the weight vector as shown in Equation 7.

(7) $$\dot{w_{t}}=\left[\begin{array}{l} w_{t}\\ b_{t} \end{array}\right],\dot{x}=\left[\begin{array}{l} x\\ 1 \end{array}\right]$$

Including the bias term into the weight vector results in a regularization of the bias, which can be a problem if a larger bias is required. Furthermore, the similarity between the tasks is facilitated by regularizing the task specific weights. Given two similar tasks with considerably different bias terms, the regularization can result in mainly forcing the bias to be similar and not the feature specific weights. To avoid this problem, we centered the target values *y* directly before the optimization and used the offset as bias. For high dimensional data, such as sparse chemical fingerprints, a bias term as shown in Equation 7 is often not required [26,27]. While we did not include regularized bias terms in our experiments because of the aforementioned reason, it can be profitable for GRMT if the average target values of the tasks differ substantially.

### Graph-regularized multi-task (GRMT) SVR

**Figure 3:**
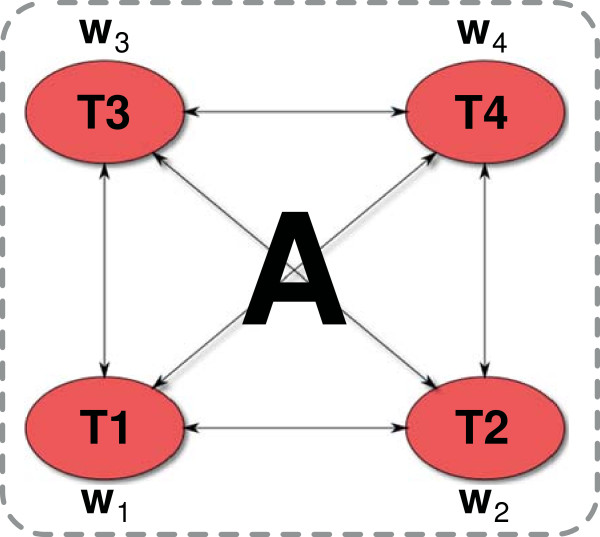
**Graph-regularized multi-task (GRMT) SVR training.** The example shows four tasks, represented by four nodes of a graph, and their corresponding weight vectors $w_{1},\ldots ,w_{4}$. The tasks are related by a real-valued adjacency matrix *A*. GRMT trains the task specific models $w_{1},\ldots ,w_{4}$ in a single step, indicated by a dashed box, using the instances of all tasks.

Evgeniou et al. introduced an approach that uses graph-based regularization [29,30]. In their approach, each task corresponds to a node in a graph and the similarity between the tasks is encoded by weighted edges summarized in an adjacency matrix *A*, where *A*_*st*_≥0 (see Figure 3). The resulting regularization $J(w_{1},\ldots ,w_{T})$ is the sum of similarity weighted distances between the weight vectors as presented in Equation 8. Using the graph Laplacian *L* = *D*−*A* of a given adjacency matrix *A*, where $D_{\text{st}}=\delta_{\text{st}}\sum_{k}A_{\text{kt}}$, the regularizer can also be expressed as shown in Equation 9.

(8) $$J(w_{1},\ldots ,w_{T})=\frac{1}{4}\sum_{s=1}^{T}\sum_{t=1}^{T}A_{\text{st}}||w_{s}-w_{t}|{|}^{2}$$

(9) $$=\frac{1}{2}\overset{T}{\underset{s=1}{\sum }}\overset{T}{\underset{t=1}{\sum }}L_{\text{st}}{w_{s}}^{T}w_{t}$$

Equation 8 indicates that the graph-regularized multi-task (GRMT) SVR strongly depends on the choice of the adjacency matrix *A*, which encodes the similarity between the tasks.

The primal GRMT SVR optimization problem is obtained by combining Equations 5 and 9, which results in the following problem.

(10) $$\begin{array}{l} \underset{w_{1},\ldots ,w_{T},\xi }{\text{min}}\;\frac{1}{2}\overset{T}{\underset{t=1}{\sum }}||w_{t}|{|}^{2}+\frac{1}{2}\overset{T}{\underset{s=1}{\sum }}\overset{T}{\underset{t=1}{\sum }}L_{\text{st}}{w_{s}}^{T}w_{t}\\ \;+C\overset{l}{\underset{i=1}{\sum }}l_{∊}(\xi_{i},y_{i})\\ \;\mathrm{s.t.}\;\xi_{i}={w_{t_{i}}}^{T}x_{i} \end{array}$$

Widmer et al. [32] proposed an alternative formulation of the primal for GRMT classification, which combines the task specific weights $w_{1},\ldots ,w_{T}$ into a single weight vector **w**. This alternative formulation uses the so-called “block vector view”. Furthermore, they proposed a new dualization technique, which allows for the derivation of a dual problem that can be optimized with an adapted version of the LIBLINEAR solver [26,27]. With the LIBLINEAR solver, the efficient training of large-scale graph-regularized multi-task problems becomes feasible.

For formulating the GRMT SVR primal problem similar to the classification formulation of Widmer et al., we first introduce the “block vector view”. The “block vector view” can be defined as shown in Equations 11 and 12, where *I*_*n*_ is the *n*-dimensional identity matrix and $L\in {\mathbb{R}}^{T\times T}$. The injective function $\psi :{\mathbb{R}}^{n}\mapsto {\mathbb{R}}^{\text{nT}}$ maps a fingerprint *x*_*i*_ to a vector that is zero, except for the *t*_*i*_-th block.

(11) $$\begin{array}{l} \text{block}(L):=\left(\begin{array}{lll} L_{11}I_{n} & \cdots & L_{1T}I_{n}\\ \vdots & \ddots & \vdots \\ L_{T1}I_{n} & \cdots & L_{\text{TT}}I_{n} \end{array}\right) \end{array}$$

(12) $$\begin{array}{l} \begin{array}{l} \psi (x_{i}):={\left(0,\ldots ,0,{x_{i}}^{T},0,\ldots ,0\right)}^{T}\\ \;↑\\ \;t_{i}-\text{th}\;\text{block} \end{array} \end{array}$$

With the “block vector view”, the primal optimization problem for GRMT SVR (10) can be reformulated as follows.

(13) $$\begin{array}{ll} \underset{w,\xi }{\text{min}} & \;\frac{1}{2}w^{T}\text{block}(I_{T}+L)w+C\overset{l}{\underset{i=1}{\sum }}l_{∊}(\xi_{i},y_{i})\\ \mathrm{s.t.} & \;\xi_{i}=w^{T}\psi (x_{i}) \end{array}$$

The dual formulation of the primal (13) can be derived with the dualization technique of Widmer et al. Details on the derivation of the GRMT SVR dual can be found in Additional file 1. The dual GRMT problem can be stated as follows.

(14) $$\begin{array}{l} \underset{\beta }{\text{min}}\;\frac{1}{2}{∥\overset{l}{\underset{i=1}{\sum }}\beta_{i}\psi (x_{i})∥}_{\text{block}(M)}^{2}\\ \;+\overset{l}{\underset{i=1}{\sum }}\left(∊|\beta_{i}|-\beta_{i}y_{i}+\frac{1}{4C}\beta_{i}^{2}\right)\\ \text{where}\;M:={\left(I_{T}+L\right)}^{-1}\\ \;\text{and}\;{∥x∥}_{B}^{2}:=x^{T}Bx \end{array}$$

Similar to GRMT classification [32], the dual problem (14) can be solved using an adapted version of the LIBLINEAR solver [26,27]. Details on the adaption of the solver can be found in Additional file 1. With the adapted LIBLINEAR solver, training a GRMT regression problem with more than 20,000 instances and over 100 tasks becomes feasible.

### Top-down multi-task (TDMT) SVR

If the learning tasks or in our case protein targets are related by some taxonomy T, the hierarchical structure of T can be exploited to subsequently train more specialized models. We assume that the longer the common evolutionary history of two targets, the more similar the structure of the proteins, and the more beneficial it should be to share information between the learning tasks. In such a taxonomy, leaves correspond to learning tasks that are related by the inner nodes.

**Figure 4:**
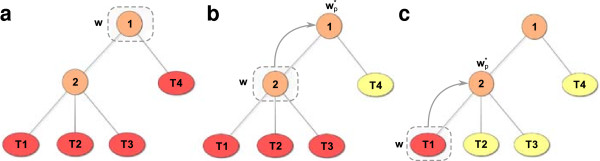
**Top-down multi-task (TDMT) training procedure.** The example shows a taxonomy with two inner nodes and four leaves or tasks. A red task indicates that the instances of the task are used for model training, whereas a yellow task means that the instances are not used for training. For each node in the taxonomy a model is trained in a top-down fashion. (**a**) First, the root model is trained taking into account all training instances. (**b**) Next, the model of the inner node 2 is trained with the instances of the subtree. The model is required to be similar to the parent model $w_{p}^{*}$ by the regularization term of Equation 15, which is indicated by a gray arrow. (**c**) Finally, the leaf model for task “T1” is trained using the instances of the task to compute the loss, while pulling the model towards the parent model. Procedure (**c**) is applied to all leaf nodes until we inferred a model for each task.

The idea of top-down multi-task (TDMT) learning is to subsequently train models for each node of the given taxonomy in a top-down manner, obtaining more specialized models while descending the taxonomy. The successive specialization is achieved by minimizing the training error with respect to the training instances of the current subtree, while maintaining similarity to the ancestor by an additional regularization term (see Figure 4). The primal optimization problem at a certain node of the taxonomy can be formulated as follows.

(15) $$\begin{array}{ll} \underset{w}{\text{min}}\; & \frac{1-B}{2}||w|{|}^{2}+\frac{B}{2}||w-w_{p}^{*}|{|}^{2}+C\underset{i\in S}{\sum }l_{∊}(\xi_{i},y_{i})\\ \mathrm{s.t.}\; & \xi_{i}=w^{T}x_{i} \end{array}$$

In Equation 15, the set S contains the training instances *i*, for which the task *t*_*i*_ is a leaf of the current subtree. The weight $w_{p}^{*}$ is the optimal weight of the parent’s SVR model, which is fixed during the optimization of the current model. The parameter *B*∈ [0,1] controls the trade-off between the margin of the current model and the similarity to the parents model $w_{p}^{*}$. Setting *B*=0 corresponds to training a model that is independent of its ancestor, whereas setting *B*=1 represents a model that is maximally dependent on its ancestor.

The primal (15) can be reformulated to the following problem.

(16) $$\begin{array}{ll} \underset{w}{\text{min}}\; & \frac{1}{2}||w|{|}^{2}-Bw^{T}w_{p}^{*}+C\underset{i\in S}{\sum }l_{∊}(\xi_{i},y_{i})\\ \mathrm{s.t.}\; & \xi_{i}=w^{T}x_{i} \end{array}$$

The alternative formulation (16) shows that the TDMT optimization problem only has an additional linear term compared to the standard SVR primal (1). Equation 17 denotes the dual optimization problem, which, limited to the set S, is also identical to the standard SVR dual *f*_*D*_(***β***) of Equation 3 except for an additional linear term.

(17) $$\underset{\beta }{\text{min}}\;f_{D}(\beta )-\underset{i\in S}{\sum }\beta_{i}\underset{p_{i}}{\underset{︸}{Bw_{p}^{*T}x_{i}}}$$

The linear terms *p*_*i*_ can be pre-computed before optimization and passed to the solver as additional linear term. Hence, the optimization problem (17) can be efficiently solved with any existing SVR solver by extending the solver to handle custom linear terms *p*_*i*_. We extended the Java port of LIBLINEAR to handle additional linear terms. As a result, the optimization of a top-down model is as fast as training an independent model. However, a top-down model for each node of the taxonomy T has to be calculated, which is more time consuming than inferring models for the leaves only.

For the prediction of an unseen data point **x**, we need to take into account the weight of the model and the weight of the parent as formulated in Equation 18.

(18) $$f(x)={\left(w+w_{p}^{*}\right)}^{T}x$$

### Task similarity parameters

Besides the standard SVR parameters *C* and *∊*, the task similarity is an essential parameter for multi-task regression. For GRMT the task similarity is encoded in the adjacency matrix *A*, whereas for TDMT the similarity is encoded in the parameter *B*. In principle, each edge *e* of the taxonomy can have a weight or distance, which results in a parameter *B*_*e*_ for each node model. Hence, the similarity information of the taxonomy can be used as parameters. For TDMT, the weights of the taxonomy are scaled to [0,1] and the parameters *B*_*e*_ are set to the scaled weights. A completely weighted taxonomy can be transformed to a distance matrix, where the distance of two taxa is the weight of the shortest path between the two taxa. To obtain a similarity matrix *A* the distance matrix is normalized to [0,1] and the distances *d* are transformed to a similarity *s*=1−*d*.

A simple approach to learn the task similarity for TDMT is based on cross-validation [22]. However, searching the best *B*_*e*_ of all nodes in a joint grid search is too expensive. A feasible approach is to do a local grid search for the best *B*_*e*_ at each node, which can be interpreted as a heuristic that limits the parameter search space based on the given taxonomy.

A problem for multi-task approaches can be negative transfer [31]. Negative transfer is knowledge transfer that results in a worse performance compared to a regression model without knowledge transfer. For the TDMT approach, it is possible to prevent negative transfer by adding the parameter *B*=0 to the grid search at the leaves to allow for an independent model, even if the parameters are given by the weighted edges of a taxonomy.

### Baseline methods

To compare the benefit of knowledge transfer of both TDMT and GRMT, we also evaluated the two baseline methods *t*SVM and 1SVM. The *t*SVM represents the usual approach whereby each of the *T* tasks stands for a single kinase and *T* independent standard regression SVMs are trained. So each of the resulting *T* models reflects solely the information provided by the corresponding kinase. For TDMT, the *t*SVM is equivalent to setting *B*=0 for all leaves. GRMT with the similarity *A*=*I*_*T*_, where *I*_*T*_ is the *T*-dimensional identity matrix, is also equivalent to *t*SVM, with the difference that the same SVR parameter *C* is used for each of the separate models.

Compared to the *t*SVM, the 1SVM represents the opposite extreme, where one model is trained on the whole kinome with the implication that all problems and all kinases are assumed to be identical. This implication is equivalent to training the root of a TDMT (see Figure 4a). Setting *A*_*st*_=1.0 for all *i*,*j* for GRMT results in a model, which is similar to 1SVM. Thus, TDMT and GRMT can be configured to be similar to both extremes and the task similarity allows for specifying from which tasks and to what extent knowledge is communicated.

### Molecular encoding

To generate the molecular fingerprints for SVR, we used the Java library jCompoundMapper developed by Hinselmann et al. [33]. With this library the extended-connectivity fingerprints (ECFP) were calculated for every compound used for training and testing. ECFPs [34] are common circular topological fingerprints that are frequently used for automatic comparison of molecules. As additional preferences we used a radius of 3 bonds (ECFP_6) and a hash space of size 2^20^ bits for the resulting hashed fingerprints. The reduction of the hash space from the standard 2^32^ bits of the ECFP to 2^20^ bits resulted in ≤0.5*%* and 4.2*%* colliding bits for the kinase subsets and the whole kinome data, respectively. Details on the hashing procedure can be found in the documentation of jCompoundMapper [33]. Additionally, we removed features that occur in more than 90% of the compounds for the whole kinome data.

A quality that speaks for the use of ECFPs is their interpretability. After training an SVM model, mappings between the hashed fingerprints and their corresponding substructure in the molecules of the training set can be established. This mapping enables a user to assign an importance to each atom and bond in a given compound. The importance can then be visualized with a heat map coloring [35]. For QSAR models, the weight of a substructure directly correlates with its activity contribution [36].

## Experimental

In this section, we first describe the data sets used for evaluation, which includes simulated as well as chemical data. Then, we present the parameters of the algorithms and the grid search ranges used for the experiments. Finally, we describe the statistical tests that were used to measure the significance of the differences between the algorithms.

### Simulated data

To analyze the behavior of multi-task regression in a controlled setting, we simulated data, varying the number of instances, the number of tasks, and the dimensionality. We adapted the simulation design of other researchers for the evaluation of multi-task classification [29,37]. Using a real-valued label instead of a class label, the design can be adopted to multi-task regression.

Each data point comprises *D* different attributes, where *D* controls the dimensionality of the data. Each attribute can adopt 6 different values, which represent an influence on the target value from very negative to very positive. The choice of each attribute is encoded by a 6-dimensional binary vector, e.g. (100000) for very positive and (000001) for very low. Thus, each data point *x*_*i*_ is a 6×*D* dimensional binary vector. The simulated data of [29,37] used only 4 attribute values, but we decided to increase the number of attribute values to better reflect the complexity of chemical fingerprints.

We generated models for *T* different tasks, each comprising *N* different training instances. The *N* training instances were sampled separately for each task. A model is encoded by a 6×*D* dimensional weight vector, where the weights were sampled attribute wise. Hence, the weight of a task *t* is a vector

$${w_{t}}^{T}=\left(w_{11},\ldots ,w_{16},\ldots ,w_{D1},\ldots ,w_{D6}\right),$$

where $\left(w_{j1},\ldots ,w_{j6}\right)$ are the weights corresponding to the *j*-th attribute. The weights of an attribute were randomly sampled from a Gaussian with mean

$$\left(-\beta ,-\frac{2}{3}\beta ,-\frac{1}{3}\beta ,\frac{1}{3}\beta ,\frac{2}{3}\beta ,\beta \right).$$

The target values *y* of the tasks were calculated using the standard multi-task prediction function (6), which means that the target values do not contain label noise.

The parameter *β* controls the noise in the data. The lower the value of *β*, the higher the noise in the data. We used *β*=3, which corresponds to a low noise in the data [29,37]. The similarity between the tasks can be controlled by varying the variance *σ*^2^ of the aforementioned Gaussian, where higher values of *σ*^2^ represent a lower task similarity. We used *σ*^2^=3*β* to model a low task similarity and *σ*^2^=0.5*β* for modeling a high task similarity, again like in [29,37]. To give an idea on how *σ*^2^ influences the task similarity, we calculated the cosine similarity (4) between the tasks for *N*=100, *T*=10, and *D*=10. A low task similarity resulted in a pairwise similarity of 0.32±0.12 between the tasks, whereas a high task similarity induced a pairwise similarity of 0.75±0.05. This similarity was reflected by a Pearson correlation between the target values of 0.43±0.14 and 0.82±0.05 for low and high task similarity, respectively.

Summarized, the toy data can be varied in the dimension *D*, the number of tasks *T*, the number of training instances per task *N*, and the similarity between the tasks *σ*^2^=*sβ*.

We calculated the task similarity for the multi-task algorithms from the weight vectors of the tasks. As taxonomy we used a tree with a root node, representing the mean of the Gaussians, directly connected to the *T* tasks. As edge weights, we used the cosine similarity between the task models and the root node model, which uses the mean of the Gaussians as attribute weights. For the GRMT approach, we directly calculated the cosine similarity between the weight vectors of the task models.

### Chemical data

For evaluating the multi-task algorithms on chemical data, we assembled a data set based on the ChEMBL database [38] with compounds against a large number of human protein kinase targets. We searched the ChEMBL database for the protein kinases of a previous study by Karaman et al., which comprises about 55% of the human kinome [39]. Karaman et al. examined the multi-kinase activity of several kinase inhibitors to assess the biological implications of their administration. The total amount of 317 kinases included 27 disease-relevant mutant variants. Of the remaining 290 distinct human protein kinases their equivalent representation in ChEMBL was identified, which resulted in 278 kinases. MYLK could not be matched, because ChEMBL only contains MLCK which is a synonym for MYLK according to UniProt [40]. The six kinases RPS6KA1 to RPS6KA6 account for 11 kinases altogether, because they are partly subdivided into N-terminal and C-terminal domain. Since ChEMBL handles this division on a lower level of the database in the description of the assays, these 11 kinases were also omitted. In general, kinase inhibitors can be classified into various types according to their binding mode, e.g. ATP-competitive and non-ATP-competitive [41,42]. These diverse types bind different locations on a kinase and therefore differ chemically from each other. Hence, different types of kinase inhibitors should be distinguished during experiments. However, it was not possible to obtain the membership of each kinase inhibitor in an automated fashion. As a result, different types of kinase inhibitors were merged.

On the basis of the 278 matched kinases all compounds were gathered for each target. Similar to the study of Hu et al. [43], all compounds had to fulfill certain criteria to be in the final data set. The first criterion was a certain ChEMBL confidence score. The ChEMBL confidence score of a compound states the confidence that the respective compound was assigned to the correct target with respect to the assay used. The highest score a compound can achieve is the value 9. Hu et al. selected compounds with a confidence score of 9 and omitted every other compound. We also allowed compounds with a score of 8 because selecting compounds with only the highest score resulted in too many data sets with an infeasible size to perform two-deep cross-validation. Additionally, the selection was restricted to molecules for which an assay type binding (B) is declared. We further excluded entries mapped to a mutant variant of a kinase, e.g. EGFR(L858R). Since the binding pockets of mutants have different amino acids available, the binding properties of compounds may differ. Therefore, only compounds mapped to the wild type were included. Like Hu et al., the final criterion for the selection was a reasonably high pIC50 value. The pIC50 value of a compound had to be at least 5.00. A pIC50 value of ≤5.00 is equal to an IC50 value of ≥10.0 *μ*m and represents a weakly active or inactive compound. Furthermore, the pIC50 or IC50 value had to be determined exactly, which excludes activity values given as relation like e.g. <50*nM* or >50*nM*. All IC50 values were converted to pIC50 values during the filtering process. Compounds with multiple pIC50 that differed more than 1 log unit where rejected to obtain a higher data precision. If this was not the case, the geometric means over all pIC50 values for the respective compounds were calculated.

We filtered compounds with undesirable, not drug-like physiochemical properties to exclude extreme outliers. We used the following specifications for this filter: 90 < Molecular Weight ≤ 900; -7 ≤ AlogP ≤ 9; Hydrogen Bond Acceptors ≤ 18; Hydrogen Bond Donors ≤ 18; Number of Rotatable Bonds ≤ 18. Additionally, structures containing non-organic atoms were discarded as well.

Due to the viability of a cross-validation, we additionally excluded 166 protein kinases, which had less than 15 compounds mapped to them. We also found 10 groups of duplicate structures with 3 compounds each, whereby 2 groups belonged to PTK2B and 8 groups to MAPK14. Since these molecules appertained to one specific kinase only, we mapped the ChEMBL ID of two structures to the third for each group. After all filtering steps we obtained 23000 compounds in total.

To reflect the experiments with the simulated data, we generated additional smaller data sets with the prerequisite that there have to be at least three kinases for every data set with an overlap of at least 85 molecules. To be more precise there has to be a pIC50 value for each of the selected kinases. As a result of these constraints, we got the four smaller data sets shown in Table 1. TK/PI3 depicts the tyrosine kinase (TK) family consisting of members from the SRC and ABl subfamily and the kinase PIK3CA of the more distant PI3/PI4-kinase family. The data of this subset comes from a study for dual inhibitors of tyrosine and phosphoinositide kinases [44]. MAPK is composed of members from the MAP kinase subfamily, also known as c-Jun N-terminal kinases, which belong to the CMGC Ser/Thr protein kinase family. The majority of the data of this subset (131 compounds) stems from 6 different studies (see ChEMBL for details), where 4 studies were conducted by the same laboratory. PIM consists of members from the PIM subfamily of the CAMK protein kinase family. Half of the data stems from one study, the majority of the remaining data points from 4 different studies. PRKC contains three members of the AGCs PKC subfamily. The data of this subset stems from many different small studies.

**Table 1:** Kinase subsets

**Identifier**	**Members**	**Size**	**Cluster sizes**
TK/PI3	HCK, PIK3CA, SRC, ABL1	123	18, 20, 39, 22, 19, 5
MAPK	MAPK8, MAPK9, MAPK10	142	32, 24, 15, 28, 21, 22
PIM	PIM1, PIM2, PIM3,	91	14, 10, 16, 17, 11, 23
PRKC	PRKCD, PRKCE, PRKCH	99	12, 10, 7, 18, 35, 16

Every compound of a subset has a pIC50 value for each kinase of the subset. The chemotype clusters were calculated with a 6-median clustering on the Tanimoto distance matrix.

Like for the simulated data, we estimated the similarity between the different tasks by calculating the correlation between the actual target values of the tasks. However, we used the Spearman coefficient instead of the Pearson correlation because the pIC50 values cannot be assumed to be normally distributed. For the TK/PI3, MAPK, PIM, and PRKC subsets we obtained Spearman correlations of 0.85-0.92, 0.67-0.85, 0.42-0.75, and 0.35-0.64, respectively. It should be noted that measuring the task similarity with a correlation measure does not capture potential differences between the average pIC50 values.

In order to evaluate the performance of the methods with respect to chemotypes, we generated a clustering on the basis of the chemical similarity between the molecules of each subset. We used a matrix with distance values based on the Tanimoto similarity and a *k*-medians clustering. On the basis of the within-cluster sum of squares we determined a suitable value of 6 for *k*. As a result, we calculated six clusters for each subset.

At last, the Standardizer was used for each data set to canonicalize and transform every molecule structure, JChem 5.12.0, 2013, ChemAxon [45] (http://www.chemaxon.com). On the basis of the guidelines by Fourches et al. [46] we used the following configuration: remove small fragments, neutralize, tautomerize, aromatize, and add explicit hydrogens. Details on the chemical data and the assigned clusters are provided in Additional file 2.

### Human kinome tree

To assess the relationships between the kinases used in our experiments, a Newick tree was generated. As a basis for this tree we used the binary dendrogram that was derived from the work of Manning et al. [24]. They built a kinome taxonomy based on the sequence similarities between the kinase domains. Each subfamily is divided in a binary fashion such that each node has two children at maximum. We also extracted the evolutionary distances of the kinases from the website http://kinase.com/human/kinome/. The content of these pages supports the published work of Manning et al. In addition to the given tree, the two atypical protein kinases RIOK1 and PIK3CA contained in our data set were directly attached to the root. As for the distances, a maximum value of 1 was chosen to reflect their low sequence similarity to all other kinases in the data set.

### Parameter settings

The task similarity for the chemical data was derived from the human kinome tree. The branch lengths of the tree were all in the range [ 0,1], as were the pairwise task distances derived from the tree, except for the two atypical kinases RIO1 and PIK3CA, which were added with a branch length of 1.0. Hence, no scaling to [ 0,1] was necessary for both TDMT and GRMT. The similarity of the atypical kinases to all other kinases was set to 0.0 for the GRMT algorithm.

The value of the regression parameter *∊* is proportional to the noise in the target values and the data set size. We evaluated the standard deviations of the IC50 values of two recent binding assays [47,48]. The IC50 values showed a relative deviation of ≈25*%*. A relative deviation of 25% amounts to a deviation in the pIC50 values of ≈0.1. Hence, we chose *∊*=0.1 as parameter value for the regression SVM. A grid search for an optimal *∊* can improve the performance of the algorithms. However, preliminary experiments did not yield substantial differences compared to *∊*=0.1 and we decided to stick with models with less parameters.

Recent publications [49,50] on the uncertainty in heterogeneous data such as ChEMBL showed that the error is usually higher than the 0.1 log units estimated in this study. The results of the studies show that the mean unsigned error is 0.44 log units for Ki data and 0.55 log units for IC50 data. These values might prove useful for estimating *∊* in future studies.

The parameters *B* and *C* were determined by a grid search. For all experiments and algorithms, except GRMT on the kinome data, we used ${\text{log}}_{2}(C)\in \{-5,-3,\ldots ,7\}$. For a large number of tasks GRMT often chose larger values for *C* because there are many weight vector combinations compared to the loss term. For GRMT on the kinome data we searched ${\text{log}}_{2}(C)\in \{2,4,\ldots ,8\}$. The grid search for the parameter *B* of TDMT used *B*∈{0,0.1,0.25,0.5,0.75,0.9,1.0}.

### Statistical analysis

In this study, the performance of an algorithm was evaluated on several random data set splits for the kinase subsets and on several cross-validation folds for the whole kinome data. All algorithms use the same training and test splits, which means that the performance values of two algorithms on a data set split can be paired. Furthermore, the performance values cannot be assumed to be normally distributed. Consequently, we used a two-sided Wilcoxon signed-rank test to decide if the performance of two algorithms differs significantly on a certain target. The significance level was set to *α*=0.05 for all tests.

On the kinase subsets, we compared multiple algorithms on a given target with each other for significant differences. Thus, we corrected the *p*-values of the Wilcoxon tests with Holm’s method [51] to control the family-wise error. On the whole kinome data, we compared a multi-task algorithm to a baseline method on all 112 kinase targets and recorded the number of significant differences. Correcting the *p*-values of the Wilcoxon test with the Benjamini and Hochberg correction [52] ensures a false discovery rate of 5% in the number of significant differences.

## Results and discussion

In this section we present the results of the five approaches *t*SVM, 1SVM, TDMTgs, TDMTtax, and GRMT on the simulated data as well as the chemical data. The chemical data can be divided into the kinase subsets and the kinome data. The TDMTgs and TDMTtax represent the TDMT algorithm, where the parameter *B* is defined by a grid search and by the taxonomy edge weights, respectively. All presented MSE performances were determined on external test data, which was not included for the training of the algorithms or the model selection.

### Simulated data

We simulated data varying the simulation parameters to capture the influence of the training set size *N*, the number of tasks *T*, the dimensionality *D*, and the task similarity on the performance of the five algorithms. We tested the following parameter ranges: For the training set size *N* we used *N*∈{15,30,45,60,75}, for the number of tasks *T* we chose *T*∈{2,4,5,10,15}, and the number of attributes *D* was set to *D*∈{6,10,14,18,22}. For each parameter setup, we generated 10 random data sets for training and testing. The generation of 10 different splits should avoid a validation bias induced by the random splitting procedure. Each test set contained 25 randomly generated test instances for each task with the same number of attributes as the training instances. Given a different number of training instances *N*, the test set stayed the same. The parameters of the algorithms were searched with a 3-fold inner cross-validation on the training set. We employed a 3-fold inner cross-validation for the model selection to ensure a test set size of ≥5.

**Figure 5:**
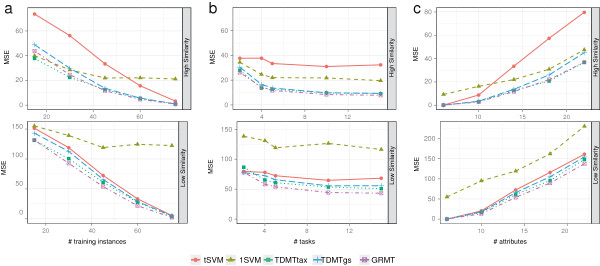
**Performance on simulated data.** Average mean squared error (MSE) while varying (**a**) the training set size *N*, (**b**) the number of tasks *T*, and (**c**) the number of attributes *D*. Varying a certain parameter, we kept the other parameters fixed to *N*=45, *T*=5, and *D*=14. The average MSE was calculated from the performance on the 10 randomly generated test data sets for each parameter setup. The upper graphs show results for high task similarity, the lower graphs for low task similarity.

The results on the simulated data with varying simulation parameters *N*, *T*, and *D* are depicted in Figure 5. The results for regression are in line with other multi-task studies on classification [22,30]. Generally, all tested algorithms except the 1SVM benefit from an increased number of training instances until the underlying problem is solved, which is reflected by an MSE close to zero. The 1SVM also benefits, but converges to a considerably higher MSE because it assumes all problems to be equal, which is not the case. The number of training instances necessary to solve the underlying problem depends on the complexity of the problem, which is controlled by the number of attributes *D*. The more attributes, the more training instances are required to solve the problem. Given similar tasks and little training data, the multi-task algorithms achieve a better MSE compared to the *t*SVM. This benefit increases with the number of tasks *T*. Overall, the benefit of multi-task algorithms compared to the *t*SVM depends on the model complexity, the number of tasks, the similarity between the tasks, and the number of training instances. Generally, the tasks have to be sufficiently similar for multi-task algorithms to benefit. Furthermore, the higher the model complexity, the higher the number of tasks, or the lower the number of training instances, the better the multi-task approaches perform compared to the *t*SVM.

Another important factor is how much additional input space is covered by the similar tasks. The multi-task approaches benefit when the tasks cover a diverging portion of the input space. If a task *s* covers a different region of the input space than a similar task *t*, knowledge can be transferred between the tasks, such that both tasks generalize well on both regions of the input space. To evaluate the influence of the additional input space coverage gained from similar tasks, we generated the same training instances for all tasks. Still, the target values *y* were different for the tasks because of the task specific models. For this simulation setup, all tasks cover the same portion of the input space and no additional coverage is achieved by transferring knowledge between the tasks. Given this setup, the multi task approaches performed equal to the *t*SVM because it is better to use the target values of the actual task than transferring knowledge from the target value of a similar task.

**Figure 6:**
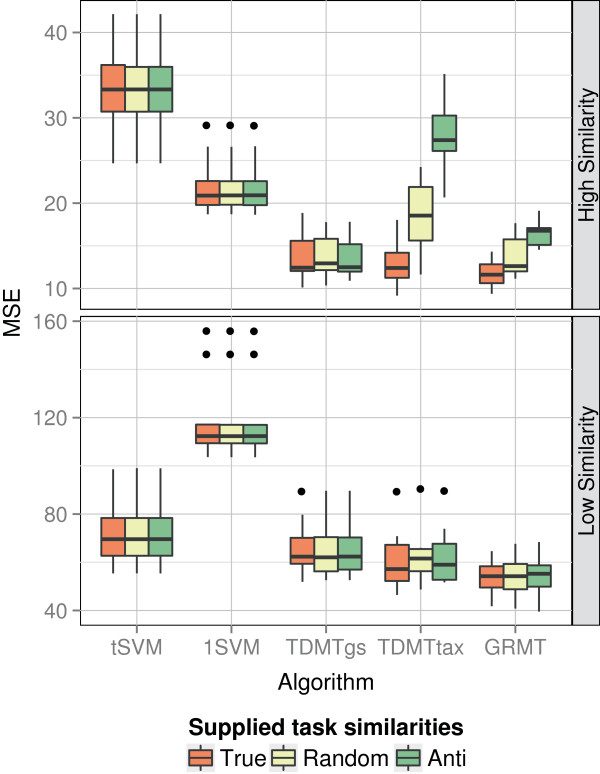
**Performance varying the supplied similarities.** Mean squared error (MSE) while varying the supplied similarities. Each boxplot visualizes the performance on the 10 randomly generated test splits. True stands for correct task similarities given by *k*_*cos*_, Anti for anti correlated similarities given by 1−*k*_*cos*_, and Random for random similarities. The upper graph shows results for a high task similarity between the simulated tasks, the lower graph for a low similarity.

Further important aspects are the influence of the task similarities supplied to the algorithms and the prevention of negative transfer. To test the impact of the supplied task similarities on the performance of TDMTtax and GRMT, we compared the true task similarities with anti correlated similarities and random similarities. The true task similarities were estimated with the cosine similarity *k*_*cos*_ between the weight vectors of the models, the anti correlated task similarities were calculated by 1−*k*_*cos*_, and the random task similarities were set to uniformly distributed random numbers from the interval [ 0,1]. The similarity of a task to itself was fixed to 1.0 for all setups. The results are depicted in Figure 6. The 1SVM, the *t*SVM, and TDMTgs do not use the supplied task similarity or determine the similarity in a grid search. Consequently, the supplied similarities did not considerably influence the performance of the algorithms. We conjecture that the small performance differences for TDMTgs are due to the randomization within the LIBLINEAR solver. For a low similarity between the simulated tasks the supplied similarity had only marginal influence, even if the algorithms were provided with anti correlated task similarities. For a high similarity between the tasks, GRMT was less prone to changes in the supplied task similarities than TDMTtax. Provided with anti correlated task similarities, the performance of TDMTtax and GRMT decreased by 120% and 40%, respectively. Thus, the task similarity is a sensible parameter for TDMTtax, whereas GRMT is more robust against changes in the supplied task similarities. It should be stated that the simulated data employed a very simple taxonomy because all tasks were direct children of the root task. Earlier studies showed, that the gain of top-down learning increases with an increasing depth of the hierarchy [53]. Hence, the simple taxonomy of the simulated data might benefit GRMT.

We tested the TDMTtax approach with and without prevention of negative transfer for all parameter combinations. We could observe a noticeable negative transfer only for simulation data with 2 tasks and a low task similarity. For the majority of simulation parameter settings, TDMTtax without negative transfer prevention achieved a better MSE. Similar results were obtained even for taxonomies with incorrect task similarities. Hence, negative transfer should not prevented for TDMTtax.

### Kinase subsets

**Figure 7:**
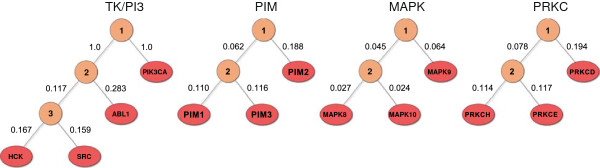
**Taxonomies of kinase subsets.** Taxonomies of the kinase subsets that were supplied to the multi-task algorithms. Each taxonomy is a subtree of the humane kinome tree.

We evaluated the five algorithms on the kinase subsets. Each subset contains only compounds that are annotated with pIC50 labels for every target of the corresponding subset. This evaluation setup allows for a controlled evaluation of the algorithms on chemical data. To obtain a different input space coverage for each task, we randomly selected 60 compounds per task. From the remaining instances of a task, we randomly chose 25 test instances, which is the reason why each subset was required to have at least 85 molecules. Compounds that are in the training set of a task are likely in a test set of a different task. Consequently, knowledge about the potency of a compound in one task can be transferred to another task provided that the tasks are sufficiently similar. We randomly generated 10 training and test sets for evaluation. For a comparable setup with respect to the simulated data, the parameter settings were determined with a 3-fold inner cross-validation. We supplied the algorithms with subtrees of the humane kinome tree that contain only targets relevant to a subset (see Figure 7).

**Figure 8:**
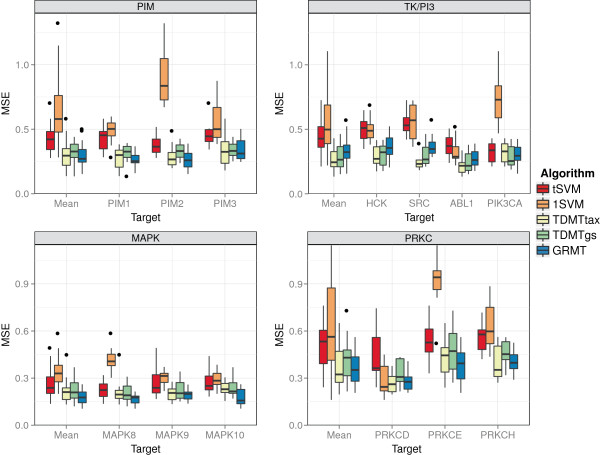
**Performance on kinase subsets.** Mean squared error (MSE) for kinase subsets. Each boxplot depicts the performance on the 10 randomly generated test sets. The target “Mean” includes the data of all targets.

The results on the kinase subsets are presented in Figure 8. Additional results, such as the performance with respect to the scaffold or when using an ECFP encoding with depth 2 (ECFP_4), can be found in Additional file 3. For all subsets, but the MAPK subset, the multi-task approaches achieved a significantly better mean performance than the baseline methods 1SVM and *t*SVM. For the MAPK and PIM set, GRMT performed best, whereas TDMTtax achieved the lowest MSE for the TK/PI3 and PRKC set. Compared to the *t*SVM baseline, the best multi-task approach decreased the MSE by 26% for the MAPK subset up to 43% for the TK/PI3 subset. Zooming in on the targets of the subsets, the performance gain of the best multi-task approach compared to the *t*SVM ranged from 16% for MAPK9 up to 56% for SRC. At least one multi-task algorithm obtained a significantly better performance than the *t*SVM for all targets except PIK3CA.

PIK3CA is part of the TK/PI3 kinase subset. The composition of this set is different compared to the other 3 subsets. While the other subsets comprise targets of the same subfamily, the TK/PI3 set contains kinases of 2 different TK subfamilies and the atypical, taxonomically distant kinase PIK3CA. However, PIK3CA is structurally similar to the eukaryontic protein kinases [24,44]. The taxonomical relationships between PIK3CA and the other 3 targets were reflected in relatively low Spearman correlations between the target values (0.35-0.45). TDMTgs could not significantly improve the performance compared to the *t*SVM for this target because of the low task similarity. GRMT and TDMTtax performed equally to the *t*SVM because the similarity to PIK3CA was set to zero by the taxonomy. Supplying GRMT and TDMTtax with the Spearman correlations resulted in a small but non-significant performance gain for both algorithms.

On the TKs ABL, SRC, and HCK the multi-task approaches improved the MSE compared to the *t*SVM. Both top-down algorithms achieved a better performance than GRMT. The 1SVM performed similar to the *t*SVM, which indicates a high similarity between the tasks. This fact was underscored by high Spearman correlations between the target values (0.85-0.92). These correlations exceed the values for the MAPK subset (0.67-0.85), although taxonomy based task similarities are low (0.43-0.67) compared to the pairwise similarities between MAPK8-10 (0.87-0.95). These results show that the kinase domain sequence similarities might not reflect the actual similarities between the pIC50 values of the training compounds. Still, the topology of the given taxonomy was reflected by the pIC50 values, which might be a reason for the promising performance of the top-down approaches. Given the high correlation between the target values, the exact value of *B* just needs to be large enough for the TK taxonomy nodes to allow for knowledge transfer between the tasks. In the given human kinome tree, even taxonomically long branches induced a similarity parameter *B*>0.5.

On the PIM subset the multi-task approaches achieved a significantly lower MSE compared to the *t*SVM for all targets. The MSE of the 1SVM is considerably higher on PIM2 than on PIM1 and PIM3. The taxonomy based task similarities indicate that PIM2 is more distantly related to PIM1 and PIM3 than they are related to each other. Additionally, inhibitors often exhibit a higher affinity against both PIM1 and PIM3 than against PIM2 [54], which is reflected by the pIC50 values of the subsets. We conjecture that the 1SVM mainly learned the structure-activity relationships based on the training data of PIM1 and PIM3, which lead to a worse performance on PIM2 because the mean pIC50 values differ by about 0.8. In contrast to the 1SVM, the multi-task approaches could exploit the taxonomy of the PIM kinases and adapt to differences in the target values, which improved the MSE. Generally, the 1SVM should achieve a high MSE when there are considerable differences in the mean pIC50 of the targets.

**Figure 9:**
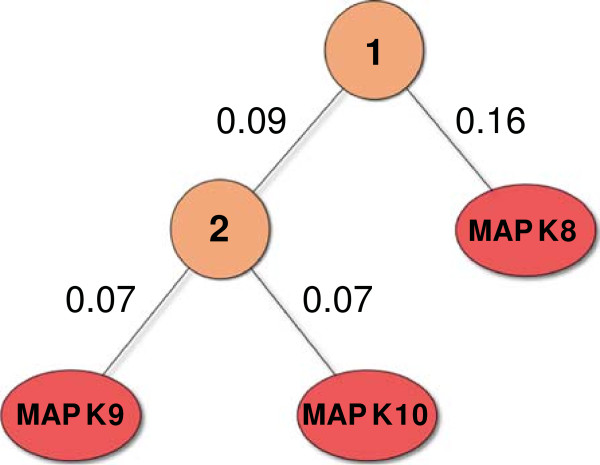
**Alternative taxonomy for the MAPK subset.** The alternative taxonomy was generated with UPGMA from the Spearman correlations between the pIC50 values of the MAPK subset targets.

For the MAPK subset, the multi-task learners achieved the smallest performance gain. The 1SVM performed considerably worse than the *t*SVM for MAPK8, which is similar to the behavior on the PIM subset. However, literature [55], the high taxonomy based task similarities (0.87-0.95), and the pIC50 values of the targets indicate a reasonably high similarity between the tasks. An explanation might be the considerably larger variance of the pIC50 values for MAPK8. The 1SVM mainly adapted to the applicability domain of MAPK9 and MAPK10, which does not include the larger pIC50 range of MAPK8. Interestingly, GRMT and TDMTgs performed significantly better than the *t*SVM on all targets of the subset, whereas TDMTtax performed similar to the *t*SVM except for MAPK9. This behavior indicates that the supplied taxonomy is suboptimal. We evaluated an alternative taxonomy, which we generated with UPGMA from the Spearman correlations between the pIC50 values. The alternative taxonomy did have slightly lower task similarities and the positions of MAPK9 and MAPK8 were swapped (see Figure 9). Supplied with this taxonomy TDMTtax also performed significantly better on MAPK8 and MAPK10 (see Additional file 3). The performance of TDMTgs also slightly increased with this alternative taxonomy on all targets but MAPK9. These results show that the topology of the taxonomy matters for top-down approaches.

On the PRKC subset, the multi-task algorithms achieved a significantly better performance than the *t*SVM on all subsets. For PRKCD, the 1SVM achieved a lower median MSE than the multi-task approaches. However, this difference was non-significant. Like on the PIM subset, the mean pIC50 of PRKCE is about 0.6 lower than the mean pIC50 of the other targets, which resulted in a high MSE for the 1SVM on PRKCE. TDMTgs performed considerably worse than TDMTtax for all targets. The pIC50 values of PRKCE and PRKCH are dissimilar compared to the similarity to PRKCD. The grid search chose *B*≤0.1 for the parent taxonomy node of PRKCE and PRKCH for 4 out of 10 repetitions. Given these parameter settings, PRKCE and PRKCH could not profit from the pIC50 value similarity to PRKCD. Furthermore, the grid search yielded *B*≤0.25 for 5 out of 10 runs for PRKCD, which resulted in a small profit for PRKCD. Optimizing both *C* and *B* resulted in overfitted parameter values for TDMTgs that do not generalize well. TDMTtax is less prone to overfitting because it only searches for *C* in a grid search.

Overall the results show that the multi-task algorithms are promising methods for inferring multi-target QSAR models. However, each of the algorithms has its drawbacks. While GRMT and particularly TDMTtax rely on sensible taxonomies, TDMTgs is prone to overfitting parameter values for small data sets.

In addition to grouping the results of a kinase subset by targets as presented in Figure 8, we grouped the results of each subset according to the clusters of a 6-medians clustering. The results (see Additional file 3) show a considerably varying MSE between the clusters of a subset. These observations indicate that the established receptor based models do not perform equal for all scaffolds as it has already been shown, e.g. by van Westen et al. [56]. Therefore, different scaffolds of our diverse multi-target set can show different performances and not every compound can be predicted equally well. Furthermore, a correlation between the size of the clusters and the performance can be observed, since scaffolds with less training instances are more difficult to predict. However, this correlation is observed for all evaluated methods and none shows a considerably stronger correlation compared to the other four. The performance on the TK/PI3 and MAPK subset is more uniform between the clusters in comparison to the PIM and PRKC subsets, which might be a result of the compilation of the data set. The binding affinities of the TK/PI3 and MAPK subsets mainly come from a few number of studies that were conducted by mainly the same laboratory, whereas the data of the PRKC subsets stems from several different studies conducted by different laboratories.

To evaluate the predictive power of multi-task learning with respect to novel targets, we performed a leave-one-sequence-out validation, which puts aside the data of a certain target for external testing while using the data of the remaining targets for training. To keep comparability to the previous setup, we used the same 25 test compounds of a target as in the previous experiments. Furthermore, the training sets had the same size as in the previous setup. To account for putting aside one target, the remaining targets received more training instances. Like before, we generated 10 different splits, which resulted in 10 different performance values per left out target.

The multi-task methods had to be adapted for the prediction of novel targets. For the TDMT approaches, the parent model of the left out target leaf was used for the prediction because a leaf model cannot be inferred without training instances. In the GRMT formulation, we adapted the graph Laplacian *L*, such that the GRMT does not regularize the model complexity (∥*w*_*t*_∥^2^) of a target *t* without training instances, but only forces the similarity to other models (*A*_*st*_∥*w*_*s*_−*w*_*t*_∥^2^).

**Figure 10:**
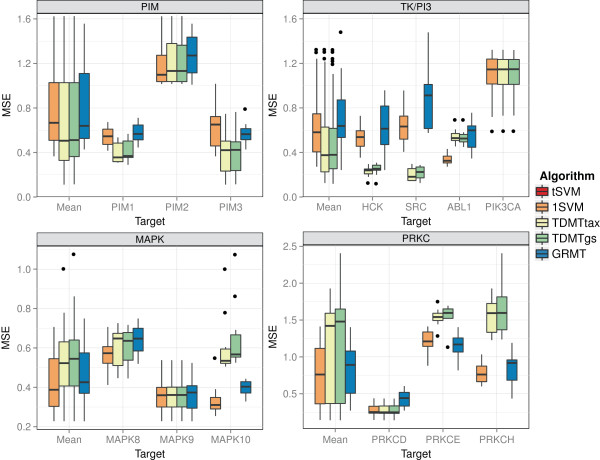
**Leave-one-sequence-out performance on kinase subsets.** Mean squared error (MSE) for leave-one-sequence-out validation. Each boxplot depicts the performance of a leave-one-sequence-out validation performed on 10 random splits. The target “Mean” includes the data of all targets. For PIK3CA, the GRMT performance was not evaluated because the task similarity to the other targets was zero.

The results of the leave-one-sequence-out experiments are depicted in Figure 10. The results show that the 1SVM exhibits a similar behavior compared to GRMT, which is different to the behavior of both top-down approaches. On 3 targets GRMT and the 1SVM perform considerably better, whereas the top-down approaches achieved a better MSE for 4 targets. Furthermore, there is always one target per subset on which the TDMT methods perform equal to the 1SVM (PIM2, PIK3CA, MAPK9, PRKCD) because the parent node of the corresponding leaf is the root, and training the root is equal to training the 1SVM. Generally, the results indicate that it is often better to train the 1SVM instead of the GRMT approach. An explanation for this behavior is, that based on the small number of targets in a kinase subset, it is better to exploit as much knowledge from the other targets as possible. For data sets with more targets and a deeper taxonomy, there might be a difference between the 1SVM and GRMT. Comparing the results to the previous evaluation setup indicates that the knowledge transfer to novel targets does only work considerably well for highly similar targets (e.g. HCK, SRC). Zooming in on the details shows that one of the main problems for the prediction of novel targets is a shift in the bias. On PIM1 and PIM3, the leave-one-sequence-out results of the TDMT algorithms are similar to the results of the previous evaluation (see Figure 8), whereas the approaches performed considerably worse for PIM2. Differences in the bias might also be the explanation for the difference between the top-down approaches and GRMT/1SVM because the TDMT methods calculate a new pIC50 bias for each node, whereas GRMT/1SVM calculate an average bias over all training instances.

### Kinome

In the final experiment, we evaluated the five algorithms on the whole kinome data using the human kinome tree as taxonomy. We assessed the performance with a 3-fold nested cross-validation that we repeated 3 times. Hence, we obtained 9 performance evaluations per algorithm and target. The data set preparation of the kinome data required at least 15 compounds for each target. Consequently, a 3-fold outer cross-validation ensures a test set size of ≥5. For the model selection, we employed a 2-fold inner cross-validation, again to ensure a test set size of at least 5.

**Figure 11:**
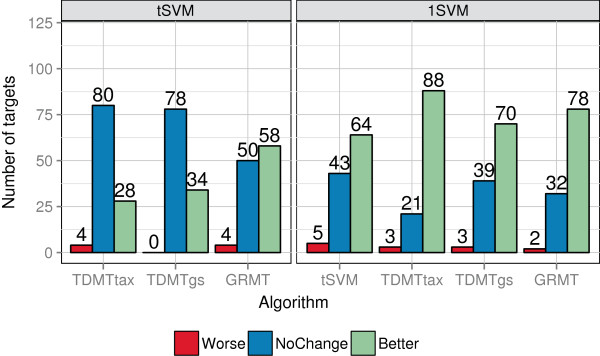
**Comparison of algorithms to baseline methods on kinome data.** Summary of the differences between each algorithm and baseline methods on the 112 kinase targets. The left graph shows the summary compared to the *t*SVM baseline, the right graph compared to the 1SVM baseline. “Worse” denotes a significantly higher MSE compared to a baseline method, “NoChange” non-significant changes, and “Better” a significantly lower MSE.

Figure 11 summarizes the results of the multi-task approaches compared to the baseline methods. Detailed results for all 112 kinase targets are depicted in Additional file 4. As to be expected, the 1SVM baseline had the worst performance on most of the data sets because the proteins of the kinome are substantially different. It obtained a considerably higher MSE on the majority of the targets. The 1SVM obtained a non-significantly different performance to the *t*SVM on 43 targets and to the multi-task algorithms on 21 targets for TDMTtax up to 39 targets for TDMTgs. On ERBB4 all other algorithms performed worse than the 1SVM. ERBB4 is a small set (39) whose compounds highly overlap with compounds of the large sets EGFR (1104) and ERBB2 (962). The overlapping molecules exhibit a high correlation between the pIC50 values (≈0.8). We think that the combination of the overlap, the high target value similarity, and possibly a restriction to a small part of the chemical space enabled the 1SVM to learn the task better than the other approaches.

Looking at the differences to the *t*SVM, GRMT performed best. It obtained a significantly lower MSE for the majority of the data sets, followed by TDMTgs, which achieved a lower MSE for a third of the targets. TDMTtax exhibited the worst performance of the multi-task algorithms and performed significantly better for only 28 targets. However, zooming in on the SRC subfamily TDMTtax achieved the best results on HCK, LYN, and YES1 and decreased the MSE by 48−75*%* compared to the *t*SVM. A similar behavior on the SRC subfamily was observed on the TK/PI3 kinase subset. The SRC subfamily tree of the human kinome taxonomy approximates the task similarities well.

TDMTgs performed at least as well as the *t*SVM on all of the targets, whereas TDMTtax and GRMT obtained a significantly higher MSE for 4 and 1 targets, respectively. The largest performance loss of GRMT amounted to 62% and was observed for MAPK3. MAPK3 is a small set (19) with a low median pIC50 (5.48) compared to the overall median pIC50 (6.7) and a low median absolute deviation (0.32). Similar to the 1SVM, GRMT centers the pIC50 values using the average over all tasks. It has to encode the bias between the average pIC50 values of the tasks using the features contained in the training compounds of the tasks. However, it might not be possible to encode the bias well, which results in a higher MSE. Thus, for taxonomically similar tasks with substantially different median pIC50 values GRMT potentially encounters difficulties. In contrast, the TDMT algorithms center the pIC50 values for each taxonomy node separately, which allows to easily adapt to changing average pIC50 values. However, this behavior results in less comparable weights between the nodes because the bias of the pIC50 values is not encoded by features of the compounds of the tasks. The problem of differing average pIC50 values between tasks can be circumvented for GRMT by adding a regularized bias term as shown in Equation 7. Another possibility is to skip the feature selection, which removes features that occur in more than 90% of the compounds. The weight of these features can act as implicit bias terms. Evaluating the performance of GRMT without feature selection resulted in a comparable performance to the *t*SVM on MAPK3 (see Additional file 4). Still, one should be cautious when using multi-task regression given tasks with considerably differing average target values.

The potency of a compound against a number of kinase targets is dependent on the structural similarity of the targets, which might be better reflected by pairwise similarities than by a taxonomy. The taxonomy forces the similarities to evolve along a tree, whereas the pairwise similarities allow for the exchange of specific structural features between the tasks. Hence, the GRMT might fit the underlying task structure more than a top-down approach. Additionally, GRMT should work well supplied with sensible pairwise similarities between protein targets. These pairwise similarities can be calculated with existing target descriptors used in proteochemometric modeling.

As shown on the simulated data, the benefit of multi-task learning depends on the model complexity, the number of training instances of a task, and the availability of a similar target. Given at least one target with sufficient similarity (≥0.8), GRMT decreased the MSE by 20% for targets with less than 100 compounds, whereas the decrease was only 6% on average for targets with at least 100 compounds. Hence, out-of-domain knowledge from other targets is mainly beneficial when not enough in-domain knowledge is available. In order to check the possible benefit of multi-task learning, we can compute a learning curve (e.g. number of compounds vs. MSE) as suggested in [22]. If the curve reaches saturation, multi-task learning is likely not beneficial. Furthermore, the benefit increases for targets with a small amount of in-domain knowledge that are similar to a target with a lot of compounds, like for YES1 in the SRC subfamily. The YES1 set comprises 37 compounds, whereas the taxonomically highly related target SRC contains 1610 compounds.

Finally, it should be mentioned that the multi-task algorithms are not designed for simultaneously inferring QSAR models on tasks as diverging as the whole kinome, but rather one should focus on a subset of desired targets.

## Conclusions

In this study, we presented two multi-task SVR algorithms and their application on multi-target QSAR models to support the optimization of a lead candidate in multi-target drug design. The first method, top-down domain adaption multi-task (TDMT) SVR, successively trains more specific models along a supplied taxonomy. For TDMT the branch lengths of the taxonomy can be supplied by the user or approximated by a grid search during training. The second method, graph-regularized multi-task (GRMT) SVR, assumes the tasks to be pairwise related with a given similarity and trains all task models in one step. The training time of both algorithms is linear in the number of training instances and tasks.

We evaluated the two TDMT SVR variants and the GRMT SVR on simulated data and on a data set of human kinases assembled from the database ChEMBL. Furthermore, we examined the behavior of the employed methods on selected subsets of the kinome data set. The results show that multi-target learning results in a considerable performance gain compared to training separate SVR models if knowledge can be transferred between similar targets. However, the performance increases only as long as not enough in-domain knowledge is available to a task for solving the underlying problem. Generally, QSAR problems are complex and high dimensional such that a considerable performance gain is apparent as long as there is sufficient similarity between the tasks, which, in particular, is the case for the kinase subfamilies. Yet, if the tasks are too similar it can be worthwhile to regard the models as identical and train a simple SVM with all data, as done by the 1SVM.

Another important aspect is the chemical space spanned by the different tasks. The lower the overlap of the chemical space spanned by the different tasks, the more multi-task learning benefits because it can transfer knowledge from different regions of the chemical space between the tasks. In contrast, if all tasks contain the same compounds, multi-task learning will not exhibit a benefit compared to training separate models because it is better to use the actual potency of a compound against a target than to transfer knowledge from a similar target. Multi-task learning is most beneficial given a task with few training compounds that is similar to a number of tasks with many training compounds, which span a diverging region of the chemical space.

Each of the presented multi-task SVR algorithms and variants has advantages and drawbacks. TDMTtax and GRMT rely on a sensible taxonomy and task similarities, respectively. Supplied with a bad taxonomy or incorrect task similarities both algorithms exhibited a considerably worse MSE on the simulated data. On the simulated data GRMT emerged to be more robust than TDMTtax, whereas both were equally robust on the chemical data. TDMTgs does a grid search for the branch lengths of the taxonomy. Thus, it only relies on the topology of the given taxonomy, which results in a robustness against suboptimal branch lengths. On the other hand, the grid search is vulnerable to overfitting parameter values, especially for small data sets.

To conclude, we think multi-task learning is a valuable approach for inferring multi-target QSAR models to help in the optimization of lead candidates. While a single-target model for each target can be used to predict multi-target binding affinities or selectivity profiles, the exploitation of the targets’ taxonomy with multi-task learning can significantly increase the quality of the predictions. In principle, the multi-task methods, particularly the top-down approaches, are able to predict novel targets if the novel target is highly similar to at least one known target and if the average pIC50 values between the targets do not differ substantially.

A focus of future studies might be the application of multi-task learning in virtual screening and the combination of our methods with the approach of Heikamp et al. [19]. Our methods can be used to infer more accurate task specific models by exploiting task similarities. Then, the accurate models can be linearly combined to search for compounds with a desired activity profile. Furthermore, the presented methods infer linear models based on the ECFPs, similar to a previous study [35]. In principle, the methods should be interpretable in a similar fashion, which can be exploited to reveal structural features that are important for binding a number of desired targets.

All multi-task learning algorithms were implemented in an in-house Java based machine learning library. The source code of the complete library is available upon request.

## Competing interests

The authors declare that they have no competing interests.

## Authors’ contributions

LR implemented the multi-task learning methods into the in-house learning library, generated the toy data, constructed the phylogenetic tree, wrote the manuscript, participated in the design of the experiments and the discussion of the results. AD assisted in the implementation of the multi-task learning methods, constructed and processed the chemical data sets, constructed the phylogenetic tree, wrote the manuscript, participated in the design of the experiments and the discussion of the results. MB and FB assisted in the construction and the filtering of the data sets, and participated in the discussion of the results. AZ supervised the study, participated in the design of the experiments, and discussed the results. All authors read and approved the final manuscript.

## Supplementary Material

Additional file 1 **Theoretical derivations of the GRMT.** This PDF document contains additional information on the theoretical derivations of the GRMT SVR dual problem and the optimization technique used to solve the problem. Click here for file

Additional file 2 **Details for the chemical data sets.** The ZIP archive contains the ChEMBL IDs and the pIC50 values of the four kinase subsets and the kinome data. A pIC50 value of “NaN” denotes a missing pIC50 value for a target and compound. Additionally, the archive contains the Newick trees for the chemical data, the task similarity matrices used for GRMT, and the size of the target sets of the kinome data. Click here for file

Additional file 3 **Additional result plots for the kinase subsets.** The ZIP archive contains results of additional evaluations performed on the kinase subsets. It includes boxplots for the chemotype specific performance on the kinase subsets, for the alternative taxonomy of the MAPK subset, and for an evaluation with ECFP encoding with depth 2 for all kinase subsets. Click here for file

Additional file 4 **Additional result plots for the whole kinome data.** The ZIP files contains PDF documents that depict the detailed results of the kinome experiments with the described setup and with a setup without feature selection. Each figure shows two bar diagrams that visualize the MSE and Q^2^ of the five algorithms on all 112 protein kinases. Click here for file
