# Supplementary material for: Inferring multi-target QSAR models with taxonomy-based multi-task learning
Source: J Cheminform. 2013 Jul 11;5:33. doi: 10.1186/1758-2946-5-33 (PMC4104930; doi:10.1186/1758-2946-5-33)
Supplement: Additional file 1 — Theoretical derivations of the GRMT. This PDF document contains additional information on the theoretical derivations of the GRMT SVR dual problem and the optimization technique used to solve the problem. [file 1758-2946-5-33-S1.pdf]

Graph regularized multi-task support vector regression

This document gives additional details on the derivation of the optimization problems for graph-regularized multi-task (GRMT) support vector regression (SVR) and shows how the problem formulations can be solved in linear time using an adopted version of the solver of the LIBLINEAR library [2, 3].

Derivation of the dual optimization problem

For the calculations we are given a multi-task data set of instances $\{(\mathbf{x_i}, y_i, t_i), i = 1, \ldots, l\}$, where $\mathbf{x_i}$ are the features of a training instance, y_i its target value, and $t_i \in \{1, \ldots, T\}$ indicates to which task the instance belongs to.

The formulation of GRMT for SVR is highly similar to the classification formulation presented by Widmer et al. [4], which results in a highly similar derivation. For the readers convenience, we do not only mention the differences but present the formulations presented in [4] including the modifications for regression.

The differences compared to classification require a different loss function and slightly different constraints which are the result of a differing prediction function. While classification SVMs predict the signed distance of a data point $\mathbf{x_i}$ to a decision surface $\mathbf{w}$ by $y_i \mathbf{w}^T \mathbf{x_i}$, regression SVMs predict a target value y_i with a regression line $\mathbf{w}^T \mathbf{x_i}$. Additionally, regression exchanges the L_2 hinge loss $l_h(\xi) = \max(1 - \xi, 0)^2$ with the ϵ-insensitive loss $l_\epsilon(\xi, y_i) = \max(|\xi - y_i| - \epsilon, 0)^2$.

Adjusting the classification primal of [4] we obtain the following primal (1), where we made use of the "block vector" and "block matrix" view shown in Equations 2 and 3,respectively.

$$\begin{aligned} \min_{\mathbf{w}, \boldsymbol{\xi}} \quad & f_P(\mathbf{w}, \boldsymbol{\xi}) = \min_{\mathbf{w}, \boldsymbol{\xi}} \quad \frac{1}{2} \mathbf{w}^T block(I_T + L) \mathbf{w} + C \sum_{i=1}^{l} l_\epsilon(\xi_i, y_i) \\ \text{s.t.} \quad & \xi_i = \mathbf{w}^T \psi(\mathbf{x_i}) \end{aligned} \tag{1}$$

$$block(L) := \begin{pmatrix} L_{11} I_n & \cdots & L_{1T} I_n \\ \vdots & \ddots & \vdots \\ L_{T1} I_n & \cdots & L_{TT} I_n \end{pmatrix} \tag{2}$$

$$\begin{aligned} \psi(\mathbf{x_i}) := (0, \ldots, 0, & \mathbf{x_i}^T, 0, \ldots, 0)^T \\ & \uparrow \\ & t_i - th\, block \end{aligned} \tag{3}$$

The derivation of the dual formulation combines Lagrangian dualization, which is common to SVM problems, with Fenchel-Legendre conjugates. Hence, the first step requires the Lagrange dualization as shown in the following Equation. The equality constraints of (1) are included in the problem formulation

Table 1: **Known fenchel conjugates.**

	loss/reg. $f(\mathbf{x})$	dual loss/reg. $f^*(\mathbf{z})$	Reference
ϵ-loss	$l_\epsilon(x, y)$	$zy + \epsilon\lvert z\rvert + \frac{1}{4}z^2$	[1]
quadratic form	$\frac{1}{2}\lVert\mathbf{x}\rVert_B^2 = \frac{1}{2}\mathbf{x}^T B\mathbf{x}$	$\frac{1}{2}\lVert\mathbf{z}\rVert_{B^{-1}}^2 = \frac{1}{2}\mathbf{z}^T B^{-1}\mathbf{z}$	[4]

by adding the constraints with the Lagrange multipliers $\beta_i \in \mathbb{R}$.

$$\begin{aligned}
\max_{\mathbf{w},\boldsymbol{\xi}} \quad - f_P(\mathbf{w},\boldsymbol{\xi}) &\overset{\text{Lagr.}}{=} \min_{\boldsymbol{\beta}} \quad \max_{\mathbf{w},\boldsymbol{\xi}} \Bigg[-\frac{1}{2}\mathbf{w}^T block(I_T + L)\mathbf{w} \\
&\qquad -C\sum_{i=1}^{l} l_\epsilon(\xi_i, y_i) - \sum_{i=1}^{l} \beta_i \left(\xi_i - \mathbf{w}^T\psi(\mathbf{x_i})\right)\Bigg] \\
&= \min_{\boldsymbol{\beta}} \Bigg[C\sum_{i=1}^{l} \max_{\xi_i} \left(-\frac{\beta_i \xi_i}{C} - l_\epsilon(\xi_i, y_i)\right) \\
&\qquad + \max_{\mathbf{w}} \left(\sum_{i=1}^{l} \beta_i \mathbf{w}^T\psi(\mathbf{x_i}) - \frac{1}{2}\mathbf{w}^T block(I_T + L)\mathbf{w}\right)\Bigg] \\
&= \min_{\boldsymbol{\beta}} \max_{\mathbf{w},\boldsymbol{\xi}} L(\boldsymbol{\beta},\mathbf{w},\boldsymbol{\xi})
\end{aligned} \tag{4}$$

Analogous to [4] we now make use of Fenchel conjugates to eliminate $\max_{\xi_i}$ and $\max_{\mathbf{w}}$. The Fenchel conjugate $f^*(\mathbf{z})$ of a function $f(\mathbf{x})$ is defined as $f^*(\mathbf{z}) := \sup_{\mathbf{x}} \left(\mathbf{z}^T\mathbf{x} - f(\mathbf{x})\right)$. Additionally, we define $\lVert\mathbf{x}\rVert_B^2 := \mathbf{x}^T B\mathbf{x}$.

$$\begin{aligned}
\min_{\boldsymbol{\beta}} \max_{\mathbf{w},\boldsymbol{\xi}} L(\boldsymbol{\beta},\mathbf{w},\boldsymbol{\xi}) &= \min_{\boldsymbol{\beta}} \Bigg[C\sum_{i=1}^{l} \underbrace{\max_{\xi_i} \left(-\frac{\beta_i \xi_i}{C} - l_\epsilon(\xi_i, y_i)\right)}_{(l_\epsilon(.,y_i))^*(-\frac{\beta_i}{C})} \\
&\qquad \underbrace{+ \max_{\mathbf{w}} \left(\mathbf{w}^T \sum_{i=1}^{l} \beta_i\psi(\mathbf{x_i}) - \frac{1}{2}\lVert\mathbf{w}\rVert_{block(I_T+L)}^2\right)}_{\frac{1}{2}\left\lVert\sum_{i=1}^{l}\beta_i\psi(\mathbf{x_i})\right\rVert_{block(I_T+L)^{-1}}}\Bigg] \\
&= \min_{\boldsymbol{\beta}} \left[\frac{1}{2}\left\lVert \sum_{i=1}^{l} \beta_i\psi(\mathbf{x_i})\right\rVert_{block((I_T+L)^{-1})} + C\sum_{i=1}^{l} (l_\epsilon(.,y_i))^*(-\frac{\beta_i}{C})\right] \\
&= \min_{\boldsymbol{\beta}} \left[\frac{1}{2}\left\lVert \sum_{i=1}^{l} \beta_i\psi(\mathbf{x_i})\right\rVert_{block(M)} + \sum_{i=1}^{l} \frac{1}{4C}\beta_i^2 + \epsilon\lvert\beta_i\rvert - \beta_i y_i\right] \\
&= \min_{\boldsymbol{\beta}} \quad f_D(\boldsymbol{\beta})
\end{aligned} \tag{5}$$

In Equation 5, we used the known Fenchel conjugates compiled in Table 1, the fact that $block(B)^{-1} = block(B^{-1})$, and $M := (I_T + L)^{-1}$.

Like in [4], we define "virtual weight vectors" $\mathbf{v_s}$ that can be expressed solely in terms of the support vectors of task s:

$$\mathbf{v_s} = \sum_{i \in I_s} \beta_i \mathbf{x_i}, \tag{6}$$

where $I_s = \{i | t_i = s\}$ comprises the indices of the instances of task s. These "virtual weight vectors" are important for solving the dual problem (5) with the regression solver of the LIBLINEAR library [3]. Using (6), we can reformulate (5) as follows.

$$\min_{\boldsymbol{\beta}} f_D(\boldsymbol{\beta}) = \min_{\boldsymbol{\beta}} \frac{1}{2} \sum_{s,t=1}^{T} m_{st} \mathbf{v_s}^T \mathbf{v_t} + \sum_{i=1}^{l} \frac{1}{4C} \beta_i^2 + \epsilon |\beta_i| - \beta_i y_i \tag{7}$$

Solving the GRMT SVR dual problem

For solving the GRMT SVR dual (7) we can adapt the regression solver of LIBLINEAR [3] similar to Widmer et al [4]. The solver of LIBLINEAR is a dual coordinate descent. A dual coordinate descent obtains a solution for the dual problem (5) by iteratively solving a one variable subproblem for each β_i. Thus, in each iteration we change only the i-th component of $\boldsymbol{\beta}$, denoted by $\beta_i + d$, keeping all other components fixed. Analogous to [3] we solve the adapted subproblem $\min_d g(d)$, where $\boldsymbol{\beta}$ is considered a constant vector and $g(d)$ is defined as follows.

$$\begin{aligned}
g(d) =& f_D(\boldsymbol{\beta} + d\mathbf{e_i}) - f_D(\boldsymbol{\beta}) \\
=& \frac{1}{2} \sum_{s,t=1}^{T} m_{st} \left(\mathbf{v_s} + d\mathbf{x_i}\delta_{s,t_i}\right)^T \left(\mathbf{v_t} + d\mathbf{x_i}\delta_{t,t_i}\right) \\
&+ \sum_{j=1}^{l} \frac{1}{4C} \left(\beta_j + d\delta_{ij}\right)^2 + \epsilon|\beta_j + d\delta_{ij}| - (\beta_j + d\delta_{ij})y_j - f_D(\boldsymbol{\beta}) \\
=& \frac{1}{2} \sum_{s,t=1}^{T} m_{st} \mathbf{v_s}^T \mathbf{v_t} + \sum_{j=1}^{l} \frac{1}{4C} \beta_j^2 + \epsilon|\beta_j| - \beta_j y_j - f_D(\boldsymbol{\beta}) \\
&+ d \sum_{t=1}^{T} m_{tt_i} \mathbf{v_t}^T \mathbf{x_i} + \frac{1}{2} d^2 \mathbf{x_i}^T \mathbf{x_i} + \frac{1}{4C} d^2 + \epsilon|\beta_i + d| - dy_i + \text{const.} \\
=& \epsilon|\beta_i + d| + d \left[\left(\sum_{t=1}^{T} m_{tt_i} \mathbf{v_t} \right)^T \mathbf{x_i} - y_i \right] + \frac{1}{2} d^2 \left(\frac{1}{2C} + \mathbf{x_i}^T \mathbf{x_i} \right) + \text{const.}
\end{aligned} \tag{8}$$

As in the derivations of the LIBLINEAR solver [3], we calculate derivates

$g'_p(d)$ and $g'_n(d)$ because $g(d)$ is not differentiable at $d = -\beta_i$. The derivates are

$$\begin{aligned} g'_p(d) &= \epsilon + \left(\sum_{t=1}^{T} m_{tt_i}\mathbf{v_t}\right)^T \mathbf{x_i} - y_i + \left(\frac{1}{2C} + \mathbf{x_i}^T\mathbf{x_i}\right) d \quad \text{if } d \geq -\beta_i, \text{ and} \\ g'_n(d) &= -\epsilon + \left(\sum_{t=1}^{T} m_{tt_i}\mathbf{v_t}\right)^T \mathbf{x_i} - y_i + \left(\frac{1}{2C} + \mathbf{x_i}^T\mathbf{x_i}\right) d \quad \text{if } d \leq -\beta_i. \end{aligned} \tag{9}$$

These derivates are equal to the formulations in [3], with the exception that $\mathbf{w}^T\mathbf{x_i}$ is replaced by $\left(\sum_{t=1}^{T} m_{tt_i}\mathbf{v_t}\right)^T \mathbf{x_i}$. Thus, all further calculations of [3] apply to the derivates (9) and we can use the LIBLINEAR solver by keeping track of the virtual weights $\mathbf{v_t}$ instead of $\mathbf{w}$. An analogous result was obtained for classification by Widmer et al. [4].

Representer theorem for GRMT SVR

Taking into account the differing prediction function, we obtain a representer theorem analogous to [4], which can be obtained by calculating $\nabla_{\mathbf{w}} \left(L(\boldsymbol{\beta}, \mathbf{w}, \boldsymbol{\xi})\right)$:

$$\mathbf{w} = (\mathbf{w_1}^T, \ldots, \mathbf{w_T}^T)^T = \sum_{i=1}^{l} \beta_i block(M)\psi(\mathbf{x_i}), \tag{10}$$

which, substituting the “block views“, is equivalent to

$$\mathbf{w_t} = \sum_{i=1}^{l} m_{t,t_i}\beta_i\mathbf{x_i}. \tag{11}$$

By combining (11) and the “virtual weight vectors” (6), the final task specific weights $\mathbf{w_t}$ can be calculated from the $\mathbf{v_t}$ after solving by

$$\mathbf{w_t} = \sum_{s=1}^{T} m_{s,t}\mathbf{v_s}. \tag{12}$$

References

[1] Radu Ioan Boţ and André Heinrich. Regression tasks in machine learning via fenchel duality. *Ann. Oper. Res.*, pages 1–15, 2013.

[2] Rong-En Fan, Kai-Wei Chang, Cho-Jui Hsieh, Xiang-Rui Wang, and Chih-Jen Lin. LIBLINEAR: A Library for Large Linear Classification. *J. Mach. Learn. Res.*, 9:1871–1874, 2008.

[3] Chia-Hua Ho and Chih-Jen Lin. Large-scale linear support vector regression. *J. Mach. Learn. Res.*, 14:3323–3348, 2012.

[4] Christian Widmer, Marius Kloft, Nico Görnitz, and Gunnar Rätsch. Efficient training of graph-regularized multitask svms. In P. A. Flach, T. Bie, and N. Cristianini, editors, *Machine Learning and Knowledge Discovery in Databases*, volume 7523 of *Lecture Notes in Computer Science*, pages 633–647. Springer Berlin Heidelberg, 2012.
