# Supplementary figures and images for: Inferring multi-target QSAR models with taxonomy-based multi-task learning
Source: J Cheminform. 2013 Jul 11;5:33. doi: 10.1186/1758-2946-5-33 (PMC4104930; doi:10.1186/1758-2946-5-33)

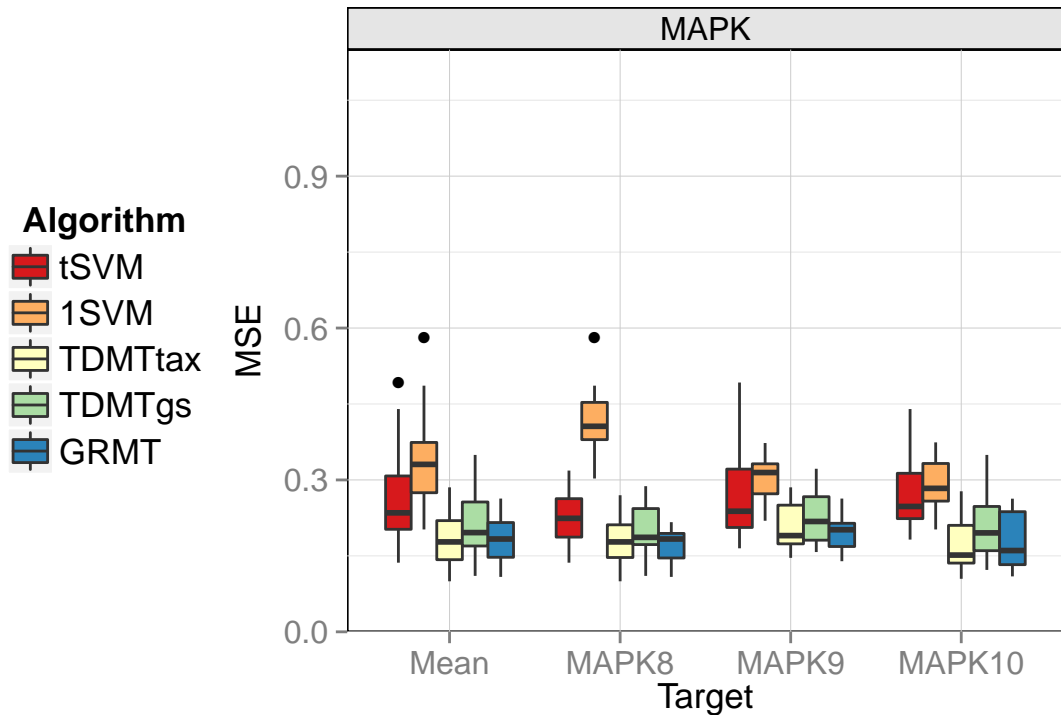

Supplement: Additional file 3 — Additional result plots for the kinase subsets. The ZIP archive contains results of additional evaluations performed on the kinase subsets. It includes boxplots for the chemotype specific performance on the kinase subsets, for the alternative taxonomy of the MAPK subset, and for an evaluation with ECFP encoding with depth 2 for all kinase subsets. [file 1758-2946-5-33-S3.zip › MAPK_AlternativeTaxonomy_ECFP6_20bit_MSE.pdf]

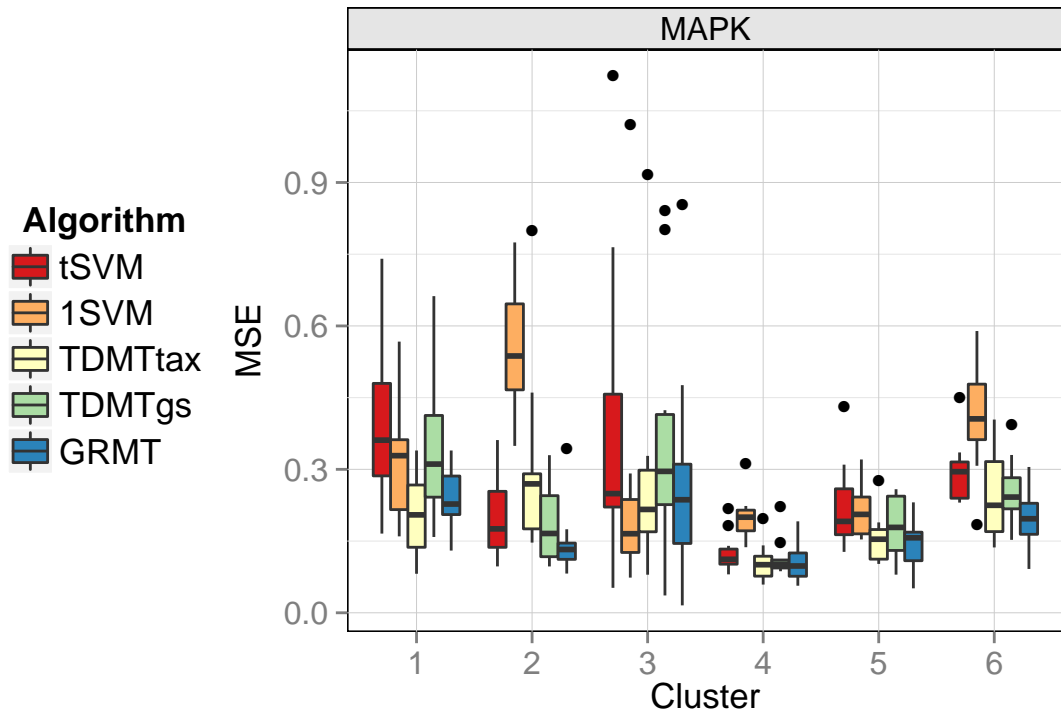

Supplement: Additional file 3 — Additional result plots for the kinase subsets. The ZIP archive contains results of additional evaluations performed on the kinase subsets. It includes boxplots for the chemotype specific performance on the kinase subsets, for the alternative taxonomy of the MAPK subset, and for an evaluation with ECFP encoding with depth 2 for all kinase subsets. [file 1758-2946-5-33-S3.zip › MAPK_cluster_ECFP6_20bit.pdf]

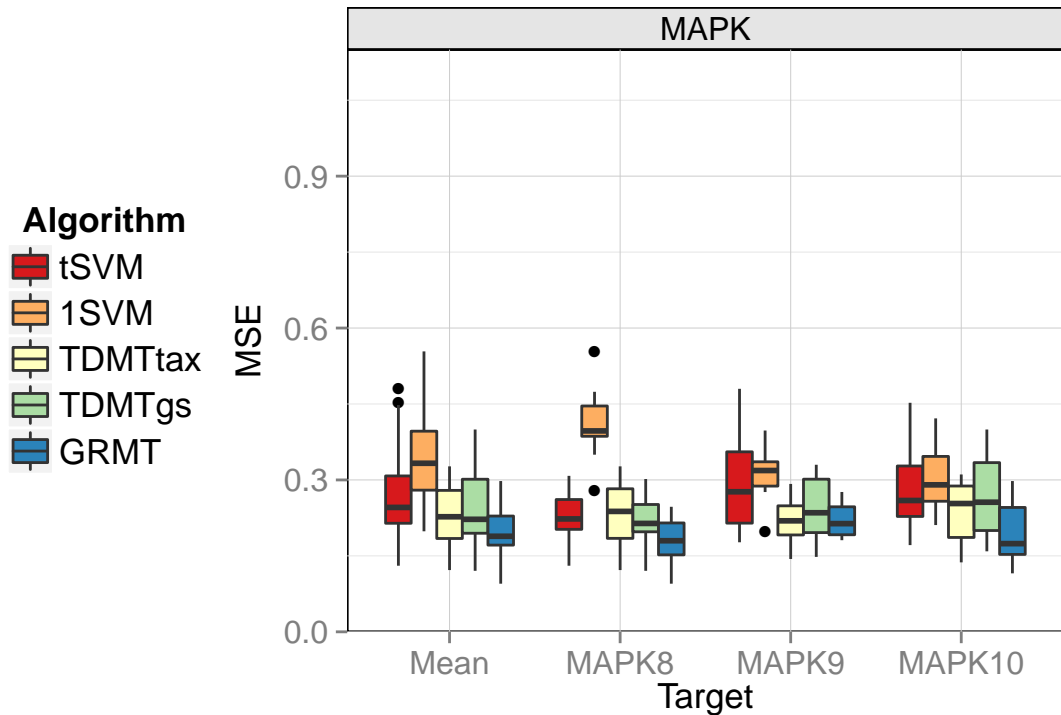

Supplement: Additional file 3 — Additional result plots for the kinase subsets. The ZIP archive contains results of additional evaluations performed on the kinase subsets. It includes boxplots for the chemotype specific performance on the kinase subsets, for the alternative taxonomy of the MAPK subset, and for an evaluation with ECFP encoding with depth 2 for all kinase subsets. [file 1758-2946-5-33-S3.zip › MAPK_ECFP4_20bit_MSE.pdf]

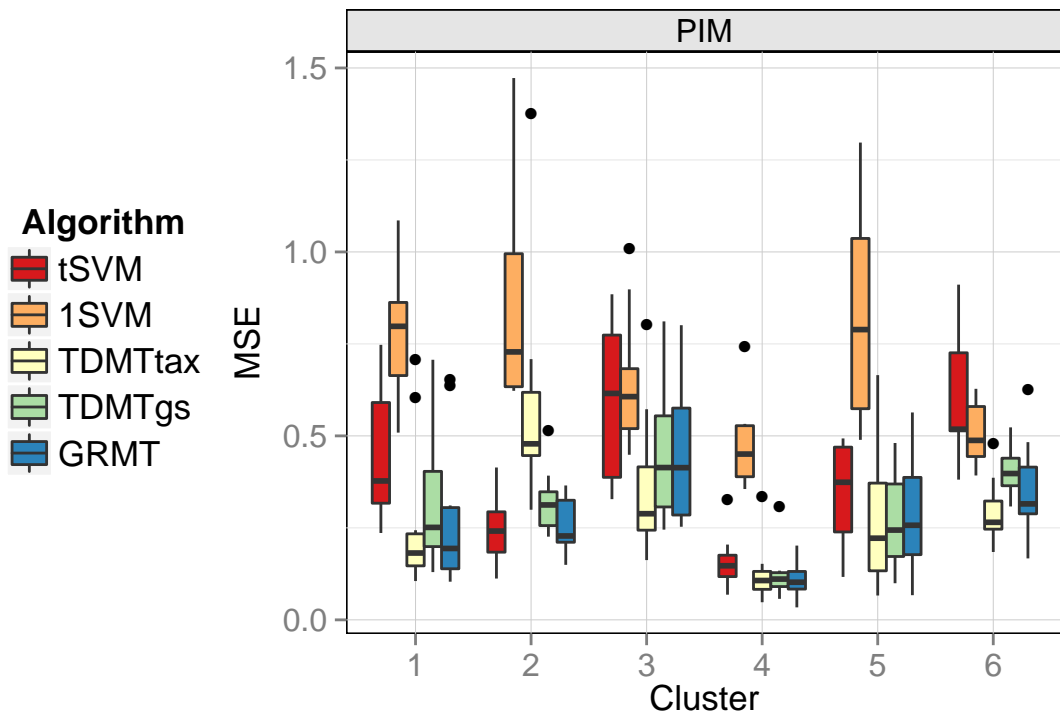

Supplement: Additional file 3 — Additional result plots for the kinase subsets. The ZIP archive contains results of additional evaluations performed on the kinase subsets. It includes boxplots for the chemotype specific performance on the kinase subsets, for the alternative taxonomy of the MAPK subset, and for an evaluation with ECFP encoding with depth 2 for all kinase subsets. [file 1758-2946-5-33-S3.zip › PIM_cluster_ECFP6_20bit.pdf]

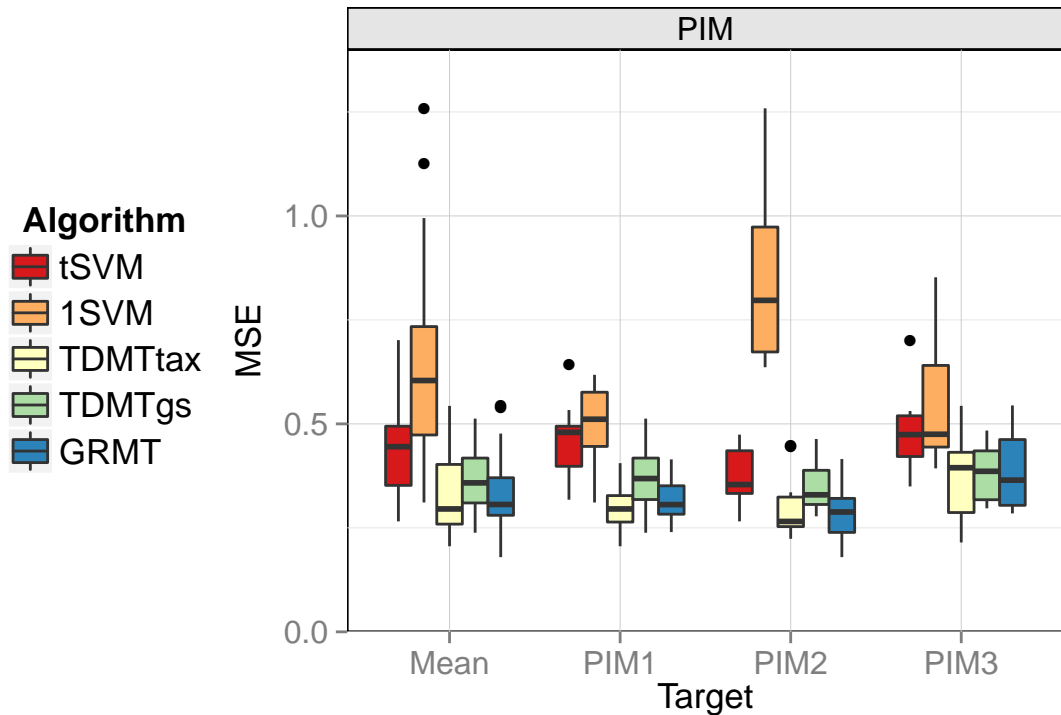

Supplement: Additional file 3 — Additional result plots for the kinase subsets. The ZIP archive contains results of additional evaluations performed on the kinase subsets. It includes boxplots for the chemotype specific performance on the kinase subsets, for the alternative taxonomy of the MAPK subset, and for an evaluation with ECFP encoding with depth 2 for all kinase subsets. [file 1758-2946-5-33-S3.zip › PIM_ECFP4_20bit_MSE.pdf]

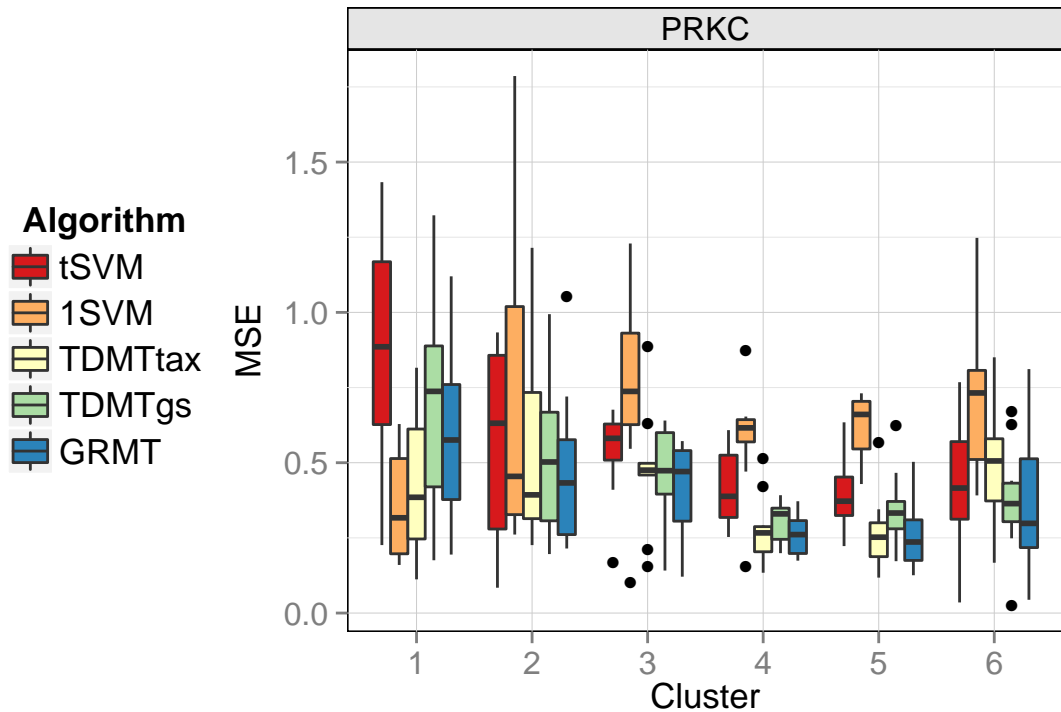

Supplement: Additional file 3 — Additional result plots for the kinase subsets. The ZIP archive contains results of additional evaluations performed on the kinase subsets. It includes boxplots for the chemotype specific performance on the kinase subsets, for the alternative taxonomy of the MAPK subset, and for an evaluation with ECFP encoding with depth 2 for all kinase subsets. [file 1758-2946-5-33-S3.zip › PRKC_cluster_ECFP6_20bit.pdf]

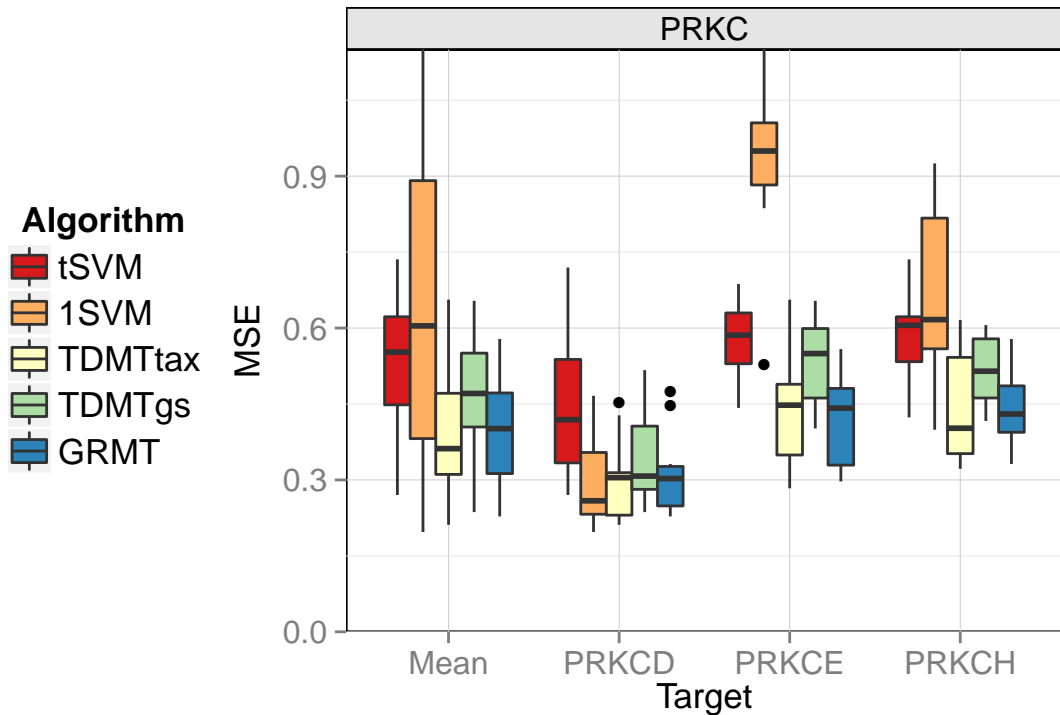

Supplement: Additional file 3 — Additional result plots for the kinase subsets. The ZIP archive contains results of additional evaluations performed on the kinase subsets. It includes boxplots for the chemotype specific performance on the kinase subsets, for the alternative taxonomy of the MAPK subset, and for an evaluation with ECFP encoding with depth 2 for all kinase subsets. [file 1758-2946-5-33-S3.zip › PRKC_ECFP4_20bit_MSE.pdf]

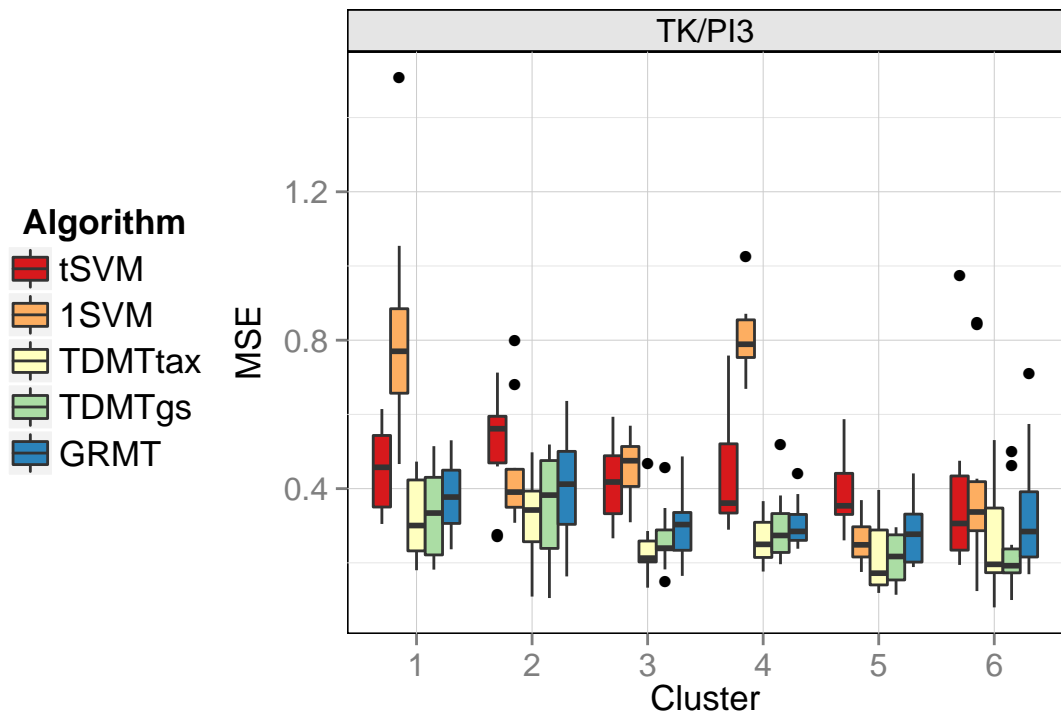

Supplement: Additional file 3 — Additional result plots for the kinase subsets. The ZIP archive contains results of additional evaluations performed on the kinase subsets. It includes boxplots for the chemotype specific performance on the kinase subsets, for the alternative taxonomy of the MAPK subset, and for an evaluation with ECFP encoding with depth 2 for all kinase subsets. [file 1758-2946-5-33-S3.zip › TK_PI3_cluster_ECFP6_20bit.pdf]

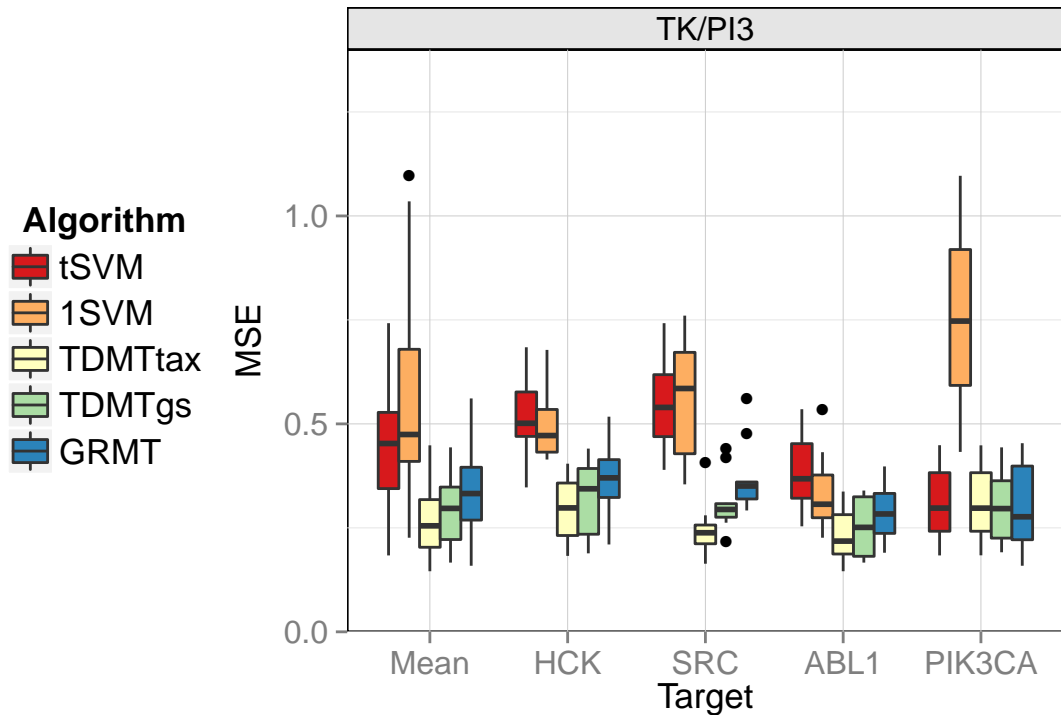

Supplement: Additional file 3 — Additional result plots for the kinase subsets. The ZIP archive contains results of additional evaluations performed on the kinase subsets. It includes boxplots for the chemotype specific performance on the kinase subsets, for the alternative taxonomy of the MAPK subset, and for an evaluation with ECFP encoding with depth 2 for all kinase subsets. [file 1758-2946-5-33-S3.zip › TK_PI3_ECFP4_20bit_MSE.pdf]

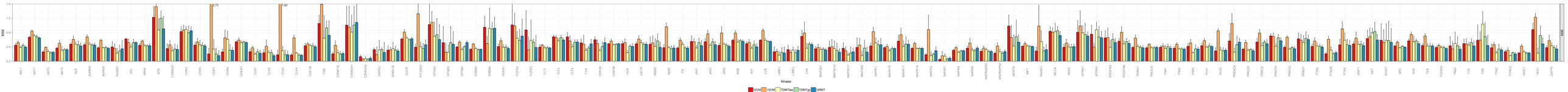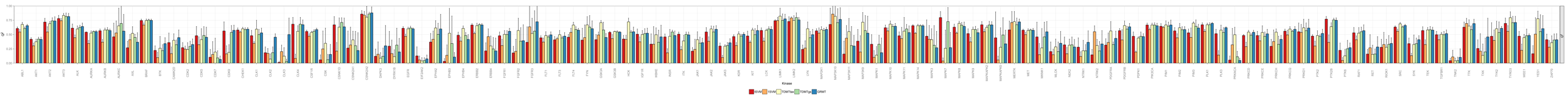

Supplement: Additional file 4 — Additional result plots for the whole kinome data. The ZIP files contains PDF documents that depict the detailed results of the kinome experiments with the described setup and with a setup without feature selection. Each figure shows two bar diagrams that visualize the MSE and Q2 of the five algorithms on all 112 protein kinases. [file 1758-2946-5-33-S4.zip › ResultsKinome_ECFP6_20bit.pdf]

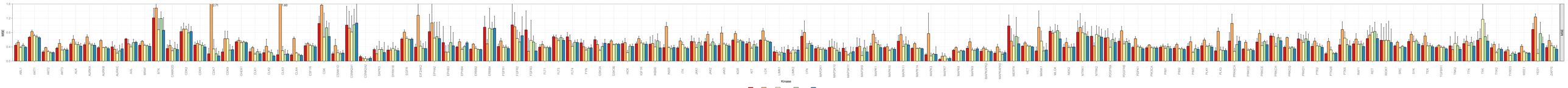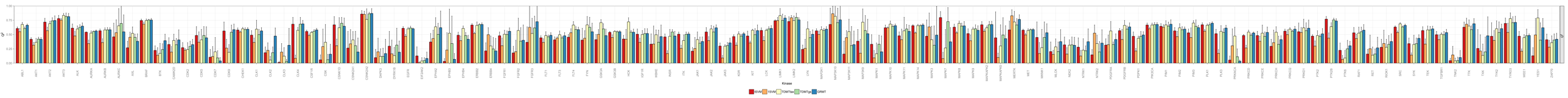

Supplement: Additional file 4 — Additional result plots for the whole kinome data. The ZIP files contains PDF documents that depict the detailed results of the kinome experiments with the described setup and with a setup without feature selection. Each figure shows two bar diagrams that visualize the MSE and Q2 of the five algorithms on all 112 protein kinases. [file 1758-2946-5-33-S4.zip › ResultsKinome_ECFP6_20bit_no_featureSelection.pdf]
